# Silver-Exchanged Zeolite Y Catalyzes a Selective Insertion of Carbenes into C–H and O–H Bonds

**DOI:** 10.1021/jacs.3c08317

**Published:** 2023-11-03

**Authors:** Yongkun Zheng, Alejandro Vidal-Moya, Juan Carlos Hernández-Garrido, Marta Mon, Antonio Leyva-Pérez

**Affiliations:** † Instituto de Tecnología Química (UPV-CSIC), Universitat Politècnica de València−Consejo Superior de Investigaciones Científicas, Avenida de los Naranjos s/n, 46022 Valencia, Spain; ‡ Departamento de Ciencia de los Materiales e Ingeniería Metalúrgica y Química Inorgánica, Facultad de Ciencias, Universidad de Cádiz, Campus Universitario Puerto Real, 11510 Puerto Real, Cádiz, Spain

## Abstract

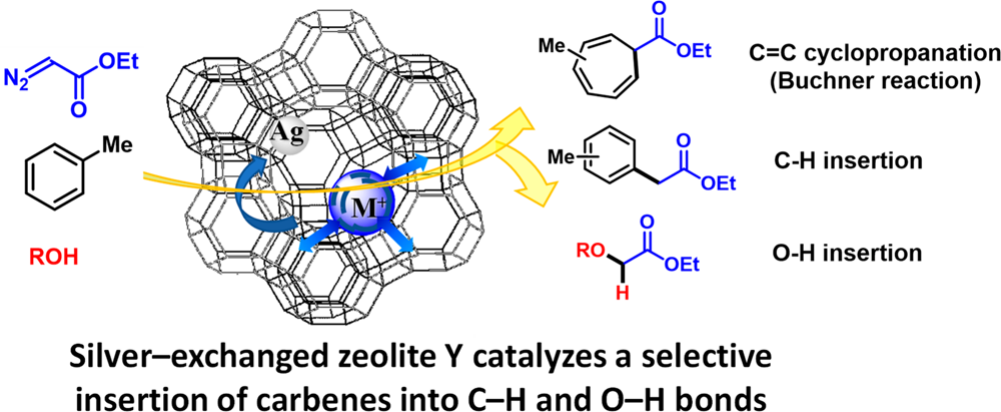

Commercially available zeolite Y modulates the catalytic activity and selectivity of ultrasmall silver species during the Buchner reaction and the carbene addition to methylene and hydroxyl bonds, by simply exchanging the counter cations of the zeolite framework. The zeolite acts as a macroligand to tune the silver catalytic site, enabling the use of this cheap and recyclable solid catalyst for the in situ formation of carbenes from diazoacetate and selective insertion in different C–H (i.e., cyclohexane) and C–O (i.e., water) bonds. The amount of catalyst in the reaction can be as low as ≤0.1 mol % silver. Besides, this reactivity allows deeply drying the HY zeolite framework by making the strongly adsorbed water molecules react with the in situ formed carbenes.

## Introduction

Ag-supported zeolites have been known for decades^1^ and found application in diverse fields such as antimicrobial agents,^2^ adsorbents of methyl iodide in nuclear power plants,^3^ and catalysts for methane activation,^4^ 1-butene dimerization,^5^ and nitrogen oxides and carbon monoxide redox reactions,^6^ among others.^7^ However, it is difficult to find in the literature the use of Ag-supported zeolites as catalysts for fine organic synthesis, despite the paramount relevance of Ag as catalyst in many organic transformations.^8,9^ The lack of examples with Ag zeolites is due to the tendency of Ag to aggregate inside the zeolite and block the pores, hampering the diffusion and reactivity of molecules composed by more than three or four atoms.^10^

In the past decade, the advent of single-atom catalysts (SACs) has spurred the investigation of solid supports to generate and stabilize SACs on surfaces, with applications in organic synthesis.^11^ In this regard, Ag is one of the more interesting chemical elements to be stabilized as a SAC, since its tendency to easily agglomerate, even with just ambient light, has severely hampered the study of the catalytic behavior of single- and few-atoms Ag entities, beyond those prepared by atom deposition or electrochemical techniques.^12^ Our group has recently reported the synthesis of Ag dimers (Ag_2_) in metal organic frameworks (MOFs) and their use as catalysts for the Buchner reaction,^13a^ the oxidation sulfonation of styrenes,^13b^ and the methanation of CO_2_.^13c^ However, single Ag atoms could not be prepared, and the electronics of the catalytically active Ag site were fixed by the MOF surroundings, without any room for catalytic tunability.

**Figure 1:**
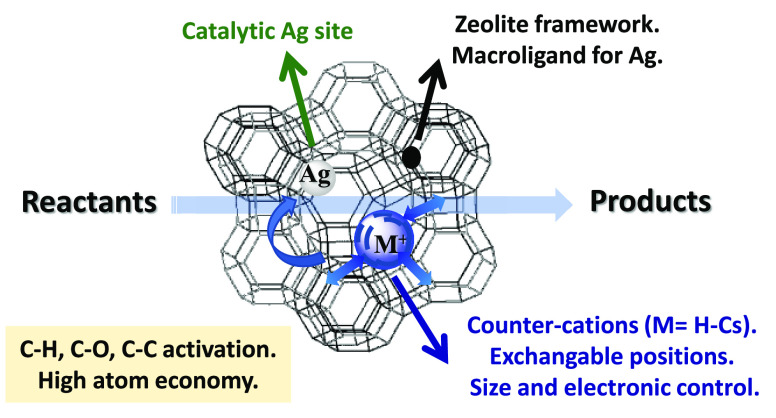
Schematic representation of the strategy employed here to prepare solid-supported Ag atoms in zeolites to be used as selective catalysts for highly efficient organic reactions. When going from H^+^ to Cs^+^, Ag receives more electron density through the zeolite framework and the steric hindrance in the supercages also increases.

Here, we show that the commercially available, robust, and crystalline microporous aluminosilicate zeolite Y stabilizes catalytically active single Ag atoms and subnanometric clusters inside its cavities, after wet exchange with AgNO_3_ and calcination under air at 450 °C. Figure 1 shows that the charge-compensating cations of the zeolite regulate the electron density on the Ag site.^14^ When going from H^+^ to Cs^+^, the electron density of the framework increases, since the bigger (softer) the counter cation, the higher the electron density on the framework, which is ultimately received by the Ag sites.^15^ In other words, a regulated electron flow occurs internally in the zeolite from the counter cation site to the Ag site, since the less electronegative counter cations, i.e. Cs^+^, enable more electron density to be received by Ag. If one also considers the difference in size between counter cations, an array of different electronic and steric Ag-supported materials, with potential application in a diversity of organic reactions, is easily obtained.

We will show here that the resulting zeolites catalyze representative carbene-mediated reactions in organic synthesis, such as the Buchner reaction and acetate insertion in methylene and hydroxyl bonds.^16,17^ The Ag-zeolite enables the reaction of C–H and C–O bonds without requiring prefunctionalized substrates or leaving groups (such as halides, etc.), and not only the catalytic activity but also the selectivity of the reaction is dictated by the counter cation of the zeolite. To our knowledge, these carbene-mediated reactions are rarely catalyzed by a solid support,^18^ and some of them are here catalyzed by Ag for the first time.

## Results and Discussion

### 1 Synthesis and Characterization of Ag-HY Zeolite

Commercially available H-USY zeolite features the lowest framework electron density among all cation-counterbalanced Y zeolites (see Figure 1); thus it was first chosen to support Ag, in order to minimize the reduction and agglomeration of the metal. Ag-HY zeolite was prepared by cationic exchange of H-USY zeolite (Si/Al = 15) with a solution of AgNO_3_ in water, to incorporate 1.08 wt % of Ag after calcination at 450 °C in air, according to inductively coupled plasma atomic emission spectroscopy (ICP-AES, Table S1 in the Supporting Information). This calcination temperature was chosen to decompose the nitrate ligands, as assessed by the lack of N in the elemental analysis (EA) of the calcined sample and also by the thermogravimetric analysis (TG, see Figure S8). It is worth commenting here that the cationic exchange method is preferred to the incipient wetness methodology since the former gives a homogeneous distribution of Ag cations on the zeolite surface and a stronger Ag–zeolite interaction, while the latter produces a heterogeneous distribution prone to facilitate the aggregation of Ag.

The resulting Ag^+^-exchanged and calcined HY zeolite (Ag-HYcal) was characterized by powder X-ray diffraction (XRD), Brunauer–Emmett–Teller surface area analysis (BET), Fourier-transformed infrared spectroscopy (FT-IR), diffuse reflectance ultraviolet visible (DR–UV–vis) and emission spectrophotometry (fluorescence UV–vis), and X-ray photoelectron spectroscopy (XPS). In some cases, for the sake of comparison, the noncalcined Ag-HY sample (just exchanged, filtered, and dried) was also measured. The XRD analysis of the calcined sample shows that the starting diffraction peaks of the HY zeolite are preserved and that any peak corresponding to Ag nanoparticles (NPs) is not observed (Figure S1). The BET analysis gives very similar surface area and microporous volume values for both HY and Ag-HYcal solids (Figure S2), and the FT-IR spectrum also confirms the integrity of the aluminosilicate composition after the calcination (Figure S3). DR–UV–vis measurements of Ag-HY, before and after calcination, show bands between 200 and 240 nm associated with few-atom Ag^+^ species,^19^ and the absence of any plasmonic band of Ag NPs (400–450 nm) for Ag-HY but only the appearance of a very small band at ∼360 nm for Ag-HYcal (Figure S4) confirms the exclusive formation of subnanometric Ag entities. The corresponding fluorescence UV–vis spectra at excitation wavelengths of 200 to 260 nm, where 2–10 Ag atom clusters should emit,^20^ show new fluorescence bands for the Ag-HY solid with respect to HY, at excitation wavelengths of 200–210 nm, with a Stokes shift of ∼150 nm, assignable to Ag_2_ and Ag_3_ clusters (Figure S5).^21^ The corresponding XPS analysis suggests only the formation of Ag^+^ sites on the HY zeolite, without extensive metallic Ag (Figure S6).^22^ A very slight shift of the Ag 3d_5/2_ toward lower values can be assigned to minor Ag^0^ species. Thus, the lack of Ag NP diffraction peaks, plasmonic bands, and fluorescence signals for >3 Ag atoms in the Ag-HY material, together with its high pore volume and XPS analysis, suggests that Ag is neither reduced nor severely aggregated within the HY zeolite and that all the supported Ag must be in cationic form.

**Figure 2:**
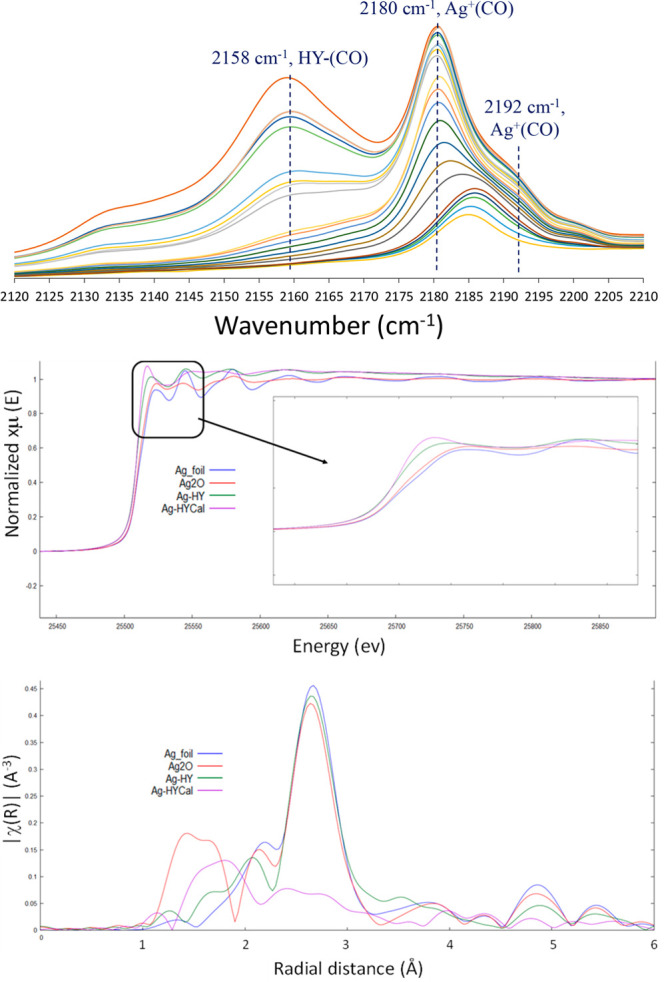
Top: Diffuse reflectance infrared Fourier transform spectroscopy (DRIFTS) of Ag-HYcal with carbon monoxide as a probe. Bottom: X-ray absorption near edge structure (XANES) and extended X-ray absorption fine structure (EXAFS) spectra of Ag-HY (purple line) compared with the uncalcined sample (green line) and Ag foil and Ag_2_O as standards (blue and red lines, respectively).

In order to further check the oxidation and aggregation state of cationic Ag^+^ in Ag-HY, diffuse reflectance infrared Fourier transform spectroscopy (DRIFTS) with carbon monoxide (CO) as a probe and also X-ray absorption spectroscopy (XAS) experiments, including X-ray absorption near edge structure (XANES) and extended X-ray absorption fine structure (EXAFS) measurements, were carried out. Figure 2 shows the results. The low-temperature DRIFTS-CO analysis shows main bands at 2180 and 2192 cm^–1^, assignable to linearly coordinated Ag^+^(CO) and Ag^+^(CO)_2_, respectively,^6c,21^ together with the expected band at 2158 cm^–1^ corresponding to the interaction between CO and the strong protons of HY zeolite.^23^ Although some minor bands can be detected at lower wavenumbers (i.e., the band at 2133 cm^–1^), it can be said here that cationic Ag species are mainly observed for the Ag-HY sample.^6c,22,24^

The XANES and EXAFS spectra, also shown in Figure 2, confirm that the Ag-HY zeolite mainly contains cationic Ag, since the spectral lines for the zeolites, either calcined or not, are more similar to Ag_2_O than to Ag foil.^13b^ The EXAFS spectra also support the concomitant formation of single Ag cations and very small Ag oxide clusters in Ag-HYcal, since the main peaks of the material appear at distances of ∼1.8 and 2.5 Å, attributable to Ag–O and Ag–Ag bonds, respectively.

With all the above characterization in hand, we must conclude that Ag-HYcal is constituted by ultrasmall cationic Ag entities, i.e., single cations and two to three Ag atom oxide clusters, supported in the unmodified HY zeolite framework.

### 2 Carbene Insertion Reactions into C–C, C–H, and O–H Bonds Catalyzed by the Ag-HY Zeolite

The new Ag-HY and Ag-HYcal zeolites were tested as catalysts for the Buchner reaction, a classical transformation in organic synthesis where a direct insertion of a carbene into the aromatic C–H bond followed by a C–C bond rearrangement occurs, which leads to otherwise very difficult to obtain cycloheptatrienes.^25^Table 1 shows the catalytic results for the reaction between ethyldiazoacetate (EDA) **1** and toluene **2** as a solvent (0.15 M), at 60 °C. For this aromatic substrate, alternatively, the insertion of the carbene into the methyl C–H bond to give the corresponding benzyl ester could also occur. The results show that the reaction does not proceed without a catalyst (entry 1), and soluble AgNO_3_ gives an 80% yield of the products (entry 2), mainly C–H insertion. The benchmark Rh_2_(OAc)_4_ catalyst for the Buchner reaction^25b^ gives a 42% yield of Buchner product **3** and 20% of C–H insertion product **4** plus dimers (35%, entry 3). Thus, it seems that AgNO_3_ is more active and more selective that Rh_2_(OAc)_4_ under the present reaction conditions. With this in mind, it could very well occur that Ag-HY is also active in the carbene-mediated reaction. Notice that products **3** and **4** are both formed after N_2_ release and carbene formation, but while product **3** comes from the cyclopropanation reaction of the triplet carbene of **1** with the C=C double bonds of toluene **2** (Buchner reaction), product **4** comes from the insertion of the singlet carbene of **1** into the C–H methyl bond of **2**. In other words, both reactions follow distinct activation mechanisms for **1**, of course directed by the electronics (and perhaps sterics) of the catalytic metal site.

**Table 1:**
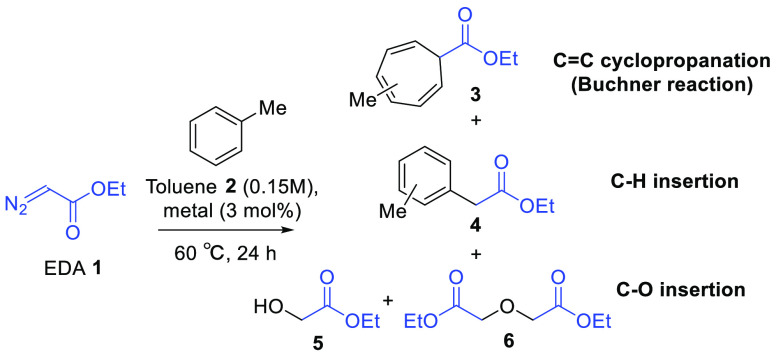
Results for the Reaction of Ethyldiazoacetate (EDA) **1** in Toluene Solvent (0.15 M) with Different Catalysts (3 mol %) under the Indicated Reaction Conditions

		product yield (%)		
entry	catalyst	**3**	**4**	**5 + 6**
1		3		
2	AgNO_3_	20	60	
3^a^	Rh_2_(OAc)_4_	42	20	–
**4**	**Ag-HY**	**5**	**14**	77 + 4
5	Ag-HYcal	5	31	50 + 12
6	Ag-HYcal and dried	6	31	50 + 11
7	Ag-HYcal (reused)	25	33	– + 42
**8**	**Ag-HYcal (6th reuse)**	**47**	**38**	– **+ 14**
9	Ag–Al_2_O_3_ (5 wt % Ag)	23	16	
10	Ag-hydrotalcite (5 wt % Ag)		5	
11	Ag-LiNaYcal	30	20	46 + –
**12**	**Ag-LiNaYcal and dried**	**55**	**42**	
13	Ag-NaYcal	14	8	48 + –
**14**	**Ag-NaYcal and dried**	**60**	**37**	
15	Ag-KNaYcal	15	5	50 + –
16	Ag-KNaYcal and dried	43	25	–
17	Ag-CsNaY	4	2	43 + –

a A 35% yield for dimers diethyl fumarate and diethyl maleate was obtained.

The noncalcined Ag-HY zeolite shows complete conversion of **1**; however, to our surprise, the main products found were **5** and **6**, presumably coming from the insertion of the carbene in the O–H bond of water present in the zeolite (entry 4),^26^ and only a 19% yield of C–H coupled products could be obtained. After calcination, Ag-HYcal shows a significant increase toward the C–C bond-forming coupled products **3** (5%) and **4** (31%); however, the main product still comes from water insertion (62%, entry 5). This result indicates that the more strongly adsorbed water molecules in the Ag-exchanged HY zeolite are acting as a reactant during the carbene reaction.

Reaction tests with 1 or 10 equiv of externally added water were carried out (Figure S7), and the results show that the reaction proceeds with complete conversion of **1** and that the product selectivity is not much varied, in other words, that external water does not participate during the O–H insertion found. Indeed, the in situ drying of the zeolite by applying vacuum at 250 °C before reaction did not improve the yield toward **3** + **4** (entry 6). Since the Ag-HYcal zeolite has lost most of the physisorbed water molecules but keeps the strongly chemisorbed water according to the corresponding TG (Figure S8), it could happen that the reuse of the zeolite will eliminate most of the O–H insertion products throughout the reuses, since chemisorbed water would become already reacted in the previous use and any external water added by the reagents would just exert a minimal influence in the reaction. In other words, the reaction of the zeolite with **1** was used as a method to deeply dry the zeolite framework of any adsorbed water molecules.

**Figure 3:**
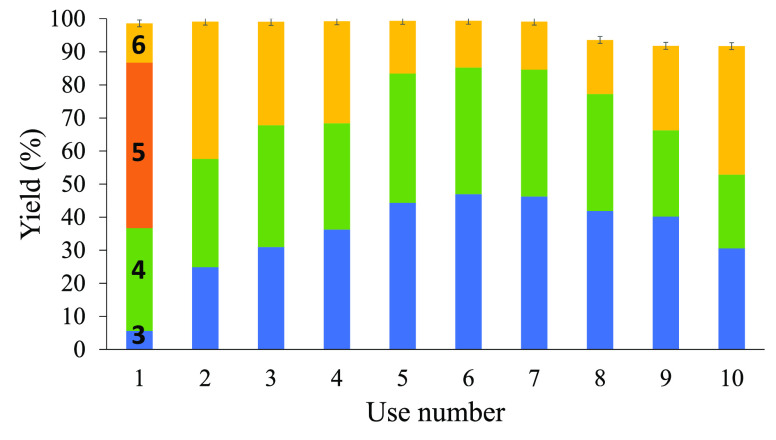
Reuses of Ag-HYcal as a catalyst (3 mol % Ag) for the reaction of ethyldiazoacetate (EDA) **1** in toluene solvent (0.15 M), at 60 °C for 24 h. Error bars account for a 5% uncertainty. See the structures of products **3**–**6** in Table 1. Zeolites were dried at 250 °C under vacuum overnight, when indicated.

Figure 3 shows that the Ag-HYcal zeolite catalyst can be reused up to seven times without depletion in catalytic activity (>90% conversion) and much better selectivity toward the C–C bond forming products **3** and **4** (>80% in uses 5–7; see also entries 7 and 8 in Table 1) as chemisorbed water reacts. It is true that the O–H insertion products start to appear again after the eighth use, which could be due to the accumulation of water in the zeolite throughout the reuses. Notice that the removal of chemisorbed water at 400 °C under vacuum overnight resulted in the zeolite color rapidly changing to brown, a much lower conversion being found when used as a catalyst, and the only products being found were those coming from water (products **5** and **6**), reflecting that some water was still there. In other words, attempts to thermally remove the chemisorbed water in the Ag zeolite only led to a severe decomposition of the catalytically active Ag species. It is also noteworthy that the typical dimerization products for **1**, i.e., diethyl fumarate and diethyl maleate, are not observed, since the isolated supported Ag sites avoid the encountering of two carbene fragments, which is an additional advantage of the supported catalyst,^25b^ while the Rh_2_(OAc)_4_ catalyst gives significant amounts of these dimers (entry 3).

Supported Ag NPs were also tested as catalysts for the reaction. For that, Ag NPs were prepared on alumina (Al_2_O_3_) and hydrotalcite (5 wt % Ag), with a very low NP average size of ∼2 nm according to high-resolution transmission electron microscopy (HR-TEM) images (Figure S9). Table 1 shows that these nanoparticulate Ag-supported species, even extremely small, are barely active for the reaction (entries 9 and 10), regardless of the support employed. It is very possible that subnanometric Ag species coexist with the ultrasmall Ag NPs on the Ag-Al_2_O_3_ and Ag-hydrotalcite solids; however, they are not active in the reaction either because of a too reduced oxidation state (Ag^0^) or because of the lack of confinement effects. In any case, with this evidence in hand, we must conclude that the ultrasmall cationic Ag species present in the HY zeolite are responsible for the catalytic activity during reaction.

### 3 Synthesis and Characterization of Ag-(Li to Cs)NaY Zeolites and Catalytic Activity for Carbene Insertion Reactions

The change in the group I counterbalancing cation of the zeolite, from H^+^ to Cs^+^, leads to an increase in the electron density of the framework, which is ultimately reflected in the electronics of the supported Ag cations.^27^ Thus, in principle, we can manipulate the electronics of the catalytic Ag site by simply changing the counterbalancing cation of zeolite Y.^15b^ Following this rationale, we prepared the different alkaline cation-exchanged Y zeolites by standard aqueous exchange of the commercially available NaY zeolite (Si/Al = 2.5) with the corresponding acetate salt solutions, and then, Ag was incorporated by the same procedure as for Ag-HY, to obtain Ag-(Li to Cs)NaY zeolites. The alkaline cation exchange values are between 5 and 50 wt %, and the Ag content is ∼1 wt % in all cases (Table S1). Calcination at 450 °C was carried out for all zeolites except for Ag-CsNaY, since Ag is rapidly reduced even after simple drying in an oven at 100 °C (dryness was performed by prolonged vacuum).

The spontaneous reduction of Ag inside the CsNaY zeolite reflects the tendency of the supported Ag cations to accept electrons from the zeolite framework, particularly from the CsNaY zeolite.^28^ In accordance with this, a comparative XRD analysis of the different Ag-Y zeolites shows the progressive appearance of the (111), (200), (220), and (311) crystallographic planes of Ag NPs as the zeolite framework gets more electron rich, from H^+^ to Cs^+^ (Figure S1).^29,30^ In any case, the aluminosilicate zeolitic structure remains stable, as assessed by FT-IR (Figure S3). The Ag-3d_5/2_ XPS signals show a very slight shift toward higher electron-binding values (Figure S6) when going from HY to CsNaY, and this shift toward higher electron-binding values is also reflected in the corresponding Si 2p (Figure S10), Al 2p (Figure S11), and O 1s (Figure S12) XPS signals.^31^ These results, together, strongly support the transference and modification of the electron density on the Ag site in the different metal-supported zeolites.

The new Ag-zeolites were tested for the reaction between EDA **1** and toluene **2**. Table 1 shows that the calcined cation-exchanged zeolite samples behave as Ag-HYcal, to give similar catalytic results (compare entries 5, 11, 13, and 15), and the corresponding kinetic results confirmed that the Ag-HYcal catalyst is the more active among them under these reaction conditions (Figure S13). However, if the Ag-zeolite catalyst is dried in situ before adding the reactants, the O–H insertion products **5** and **6** disappear and only the C–C coupling products **3** and **4** are formed, in yields of up to 97% with Ag-LiNaY and Ag-NaY (Table 1, entries 12 and 14, respectively). A hot filtration test for Ag-LiNaY shows that there is not any catalytically active species in solution (Figure S14), confirming the heterogeneous nature of the catalysis and the stability of the zeolite in reaction. In accordance, the XRD and FT-IR spectra of the used Ag-LiNaY catalyst are similar to those of the fresh zeolite sample (Figures S15 and S16, respectively). Ag-CsNaY, which shows the highest amount of Ag^0^ species and cannot be dried at >100 °C prior to reaction, gives poor catalytic results (Table 1, entry 17).

**Figure 4:**
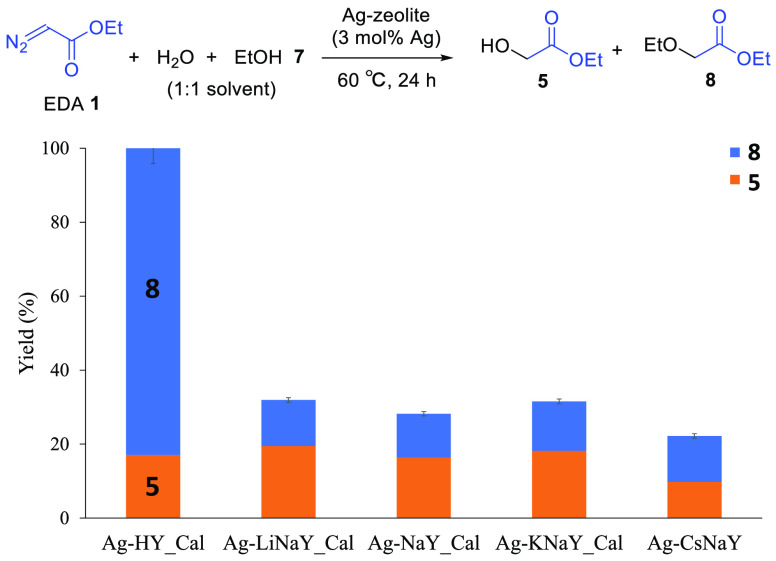
Catalytic results for the reaction of ethyldiazoacetate (EDA) **1** in a 1:1 v/v mixture of water/ethanol (0.15 M) with different Ag-zeolites as catalysts (3 mol % Ag), at 60 °C for 24 h. Zeolites were dried at 250 °C under vacuum overnight except for Ag-CsNaY. Error bars account for a 5% uncertainty.

The higher catalytic activity of the alkaline-exchanged zeolites with respect to Ag-HY toward the C–C bond-forming products **3** and **4** could be due to an intrinsic higher selectivity of the Ag catalytic site for C–C and C–H bond activation or simply to a lack of water in the zeolite once dried. However, the FT-IR spectra of the calcined solids show similar amounts of adsorbed water in all zeolites (Figure S3). In order to check if Ag-(Li to K)NaYcal zeolites are less catalytically active than Ag-HYcal toward the insertion of the carbene in the O–H bonds, the catalytic reaction of EDA **1** in a mixture of water/ethanol **7** was performed. Ethanol **7** was coadded to water in order to assess the reactivity of an external O–H bond, not present in the zeolite. The results in Figure 4 show that the Ag-HYcal catalyst gives three times more yield of O–H insertion products **5** + **8** than the alkaline zeolites; indeed, product **8** is the major product. Thus, we can conclude that the higher catalytic activity of Ag-(Li to K)NaYcal zeolites to products **3** and **4** comes from a better intrinsic selectivity of the Ag catalysts toward C–C and C–H bond activation, while Ag-HYcal prefers to activate and insert the carbene in O–H bonds. Indeed, other alcohols such as 2-chloroethanol (98% yield), phenylethanol (98% yield), allyl alcohol (97% yield), and propargyl alcohol (95% yield) engaged extremely well in the Ag-HYcal-catalyzed reaction. In summary, the counter cation nature and not the water content is the major parameter affecting the activity and selectivity of the carbene insertion reaction: while HY directs the insertion toward O–H bonds in the presence of O-containing nucleophiles (such as water and alcohols) and toward C–C and C–H bonds, equally, without water in the reaction media, alkaline-earth cations divert the insertion toward C–C and C–H bonds, in an approximately 1:2 ratio (Figure S17). These selectivity results make sense if we consider that O–H bonds are much more polar than C=C or C–H bonds; thus, the higher the electron deficiency in the Ag catalytic site (the more Lewis acid the Ag is), the better the activation of the O–H vs C=C or C–H bonds.

**Figure 5:**
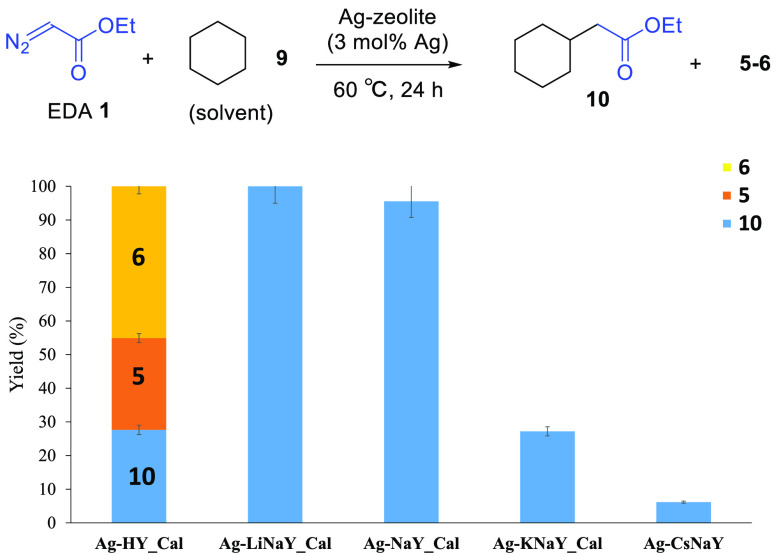
Catalytic results for the reaction of ethyldiazoacetate (EDA) **1** in cyclohexane **9** (0.15 M) with different Ag-zeolites as catalysts (3 mol % Ag), at 60 °C for 24 h. Zeolites were dried at 250 °C under vacuum overnight, except for Ag-CsNaY. Error bars account for a 5% uncertainty.

The high catalytic activity of the Ag-(Li to Cs)NaYcal zeolites to C–H insertion reactions opens the door for more challenging transformations, such as the insertion of EDA **1** in neat alkanes. Figure 5 shows that cyclohexane **9** incorporates the carbene from **1** in >95% yield with complete selectivity to product **10** on either the Ag-LiNaYcal or Ag-NaYcal catalyst. Such an excellent product yield for **10** is rarely found for any solid catalyst as far as we know.

### 4 Nature of the Catalytically Active Ag Species on Ag-LiNaY Zeolite and Detection of the Solid-Supported Ag-Carbene Species

**Figure 6:**
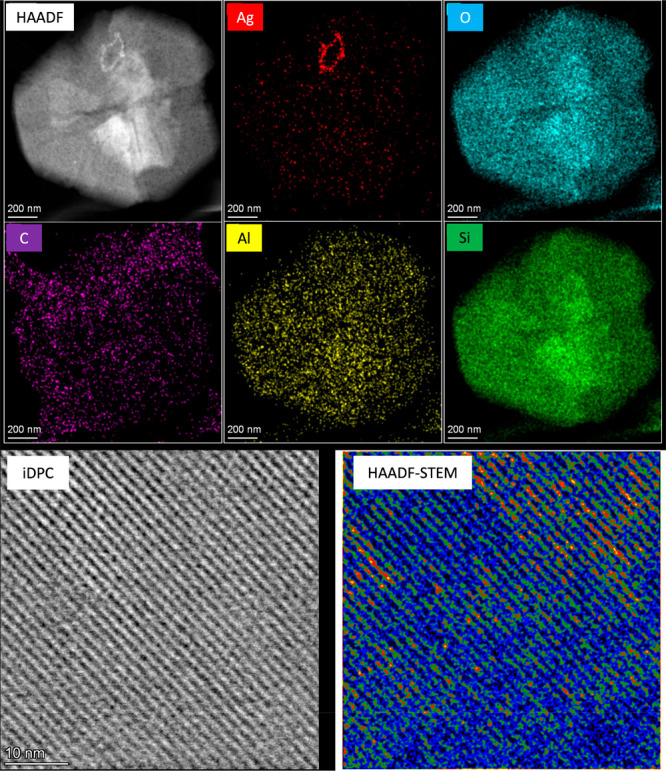
Top: Compositional mapping of a representative AC HAADF-STEM image of a Ag-LiNaYcal crystallite using EDX spectroscopy to detect the different elements. Bottom: High-resolution AC HAADF-STEM (left) and iDPC (right) images of a Ag-LiNaYcal sample. The identification of Ag entities is performed on the HAADF image due to the higher contrast and by means of iDPC images, to reveal the atomic structure of the zeolite LiNaYcal (see also Figure S19). The identification and location of the subnanometric (<1 nm) Ag entities within the zeolite framework are obtained by combining the images obtained in these two modes.

Aberration-corrected high-angle annular dark-field scanning transmission electron microscopy (AC HAADF-STEM) measurements of a Ag-LiNaYcal sample were carried out, and they are shown in Figure 6 (top). The energy-dispersive X-ray spectroscopy (EDX) mapping of a zeolite crystallite confirms the homogeneous distribution of all elements in the zeolite, including Ag (see also Figure S18), which shows a minor degree of aggregation in the form of NPs, in accordance with the XRD analysis (see Figure S1). Elemental EDX quantification fits well with the ICP-AES results (Table S1).

Figure 6 (bottom) shows aberration-corrected scanning transmission electron microscopy (AC-STEM) images of the Ag-LiNaYcal sample at 2 million times magnification. The integrated differential phase contrast (iDPC) image shows low-*Z* elements with bright contrast and dark background. The colored HAADF-STEM image can reliably identify Ag species as the brightest contrasts in these images, since HAADF-STEM imaging is proportional in good approximation to the squared atomic number, *Z*^2^. Furthermore, the visualization of the Ag entities has been improved by submitting raw HAADF images to advanced image processing, which included denoising and background subtraction (see Figure S20). In order to determine in a fully automated, user-independent, and statistically meaningful way the size of the Ag clusters observed in the experimental images, a segmentation based on K-means clustering techniques was performed on the HAADF-STEM images.^15b,32^ The application of the K-means clustering method to the experimental images reveals the major presence of Ag single atoms and ultrasmall clusters together with minor Ag NPs detected (see also Figure S21). Modeling and image simulations (Figures S22 and S23) confirm these results. All these results, together, noticeably evidence that the majority of Ag species inside the zeolite corresponds to Ag single atoms and ultrasmall Ag clusters, in good accordance with the DR–UV–vis, DRIFTS, and XAS results shown above for the Ag-HY zeolite.

**Figure 7:**
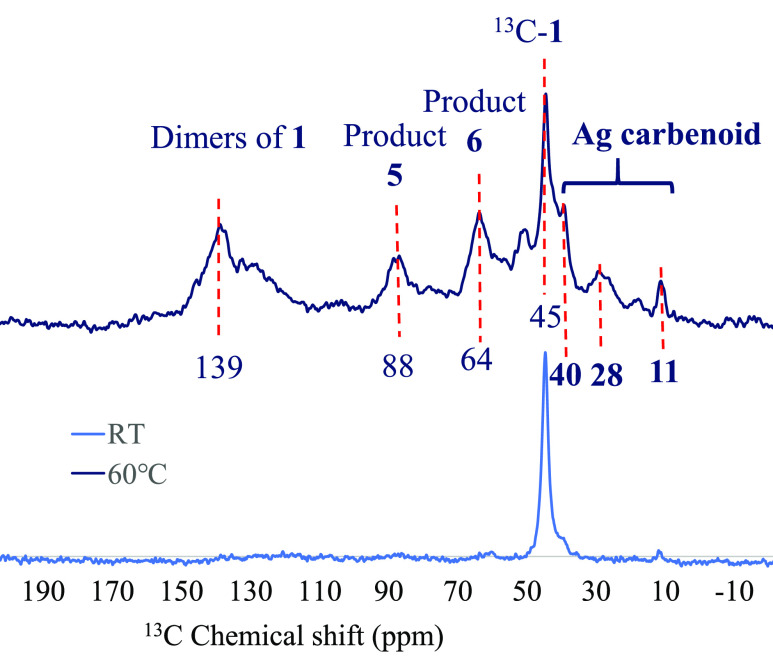
^13^C cross-polarization magic angle-spinning nuclear magnetic resonance (^13^C CP/MAS NMR) spectra of **1-**^13^C^12a^ and after reaction of EDA **1** within the Ag-LiNaYcal zeolite.

Figure 7 shows the ^13^C cross-polarization magic angle-spinning nuclear magnetic resonance (^13^C CP/MAS NMR) spectrum of adsorbed, isotopically labeled EtOOC^13^CHN_2_ (^13^C-**1**)^33^ and the resulting spectrum after sealing an ampule with EDA **1** and an equimolecular amount of Ag in Ag-LiNaYcal zeolite and making them react at 60 °C for 3 days. The CP NMR technique visualizes the carbon atoms containing C–H bonds and, thus, in our case, the carbene atom in **1**. It can be seen that the original signal of **1** at 45 ppm disappears in the presence of stoichiometric Ag, to give new upshielded signals at 11, 28, and 40 ppm, compatible with a metal carbenoid coordinated through the sp^2^ O atom of the carbonyl group to the Ag species (see Figure 9).^18a,34^ Besides, the signals corresponding to the expected products with the water molecules in the zeolite at 64 ppm (product **6**) and 88 ppm (product **5**) are also observed, as well as some dimers produced under these stoichiometric reaction conditions (139 ppm). Notice that the ester signals at ∼170 ppm are not detected because they do not contain CH bonds.

**Figure 8:**
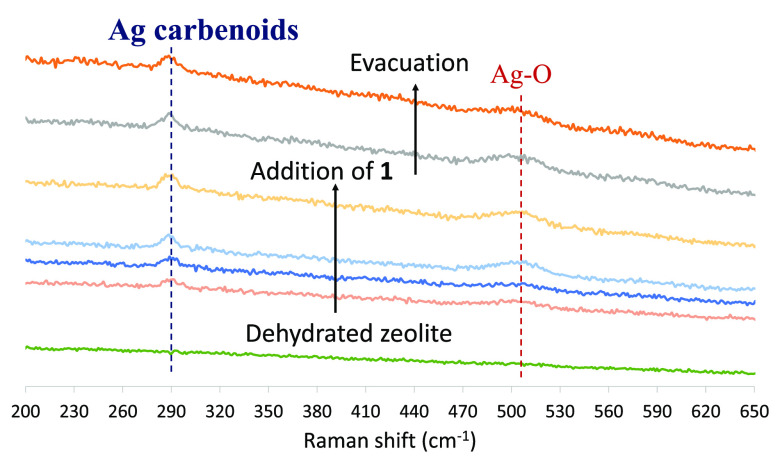
Raman spectroscopy of the Ag-LiNaY zeolite after dosing EDA **1** (four shots) and evacuation under vacuum (twice).

**Figure 9:**
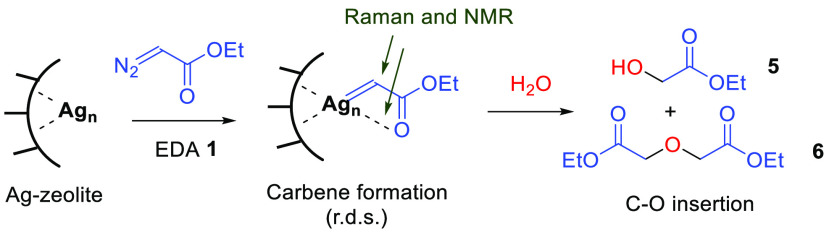
Proposed reaction mechanism for the carbene formation and water insertion on the Ag-zeolite catalyst (rds: rate-determining step), also showing the possible configuration of the Ag carbene intermediate according to the Raman and ^13^C CP/MAS NMR spectra.^18a,34^

Figure 8 shows the corresponding time**-**resolved Raman spectra of the Ag-LiNaY zeolite after dosing EDA **1**, and new bands at ∼290 and 510 cm^–1^ appear, which correspond to reported metal carbene complexes^18a^ and Ag–O bonds,^18a,21^ respectively, with the sp^2^ ester O atom coordination. These bands are stable and remain after vacuum evacuation of the sample.

In order to have more information about the reaction pathway and give the reaction mode at a molecular level, we performed kinetic analysis with increasing amounts of the different reagents (semistationary state conditions) to calculate the reaction order for each reagent and, thus, the rate equation. The results (Figure S24) show a reaction order for the Ag-HYcal zeolite catalyst, EDA **1**, and water of 1, 1, and 0, respectively. The same result for EDA **1** is found with the Ag-LiNaYcal zeolite catalyst. Therefore, the rate equation can be written as *v*_0_ = *k*_exp_[Ag-zeolite][EDA **1**], indicating that the rate-determining step (rds) of the reaction involves the formation of the Ag-carbene complex. This is the reason that the carbene intermediate is just briefly seen since, after being formed, it rapidly reacts with water to give products **5** and **6**.

With all this information in hand, the proposed mechanism for the reaction with water is shown in Figure 9, which also includes the configuration of the carbene Ag-zeolite. The first step of the process is the formation of the Ag carbene, which is the rds of the reaction. The configuration of this carbene intermediate shows that the carbonyl ester group coordinates to the metal site, as informed by the Raman and ^13^C CP/MAS NMR spectra.^18a,34^ This carbene is then inserted into the O–H bond of water to give the final product **5** (and then **6** by subsequent carbene insertion) and regenerate the starting Ag-zeolite catalyst.

**Figure 10:**
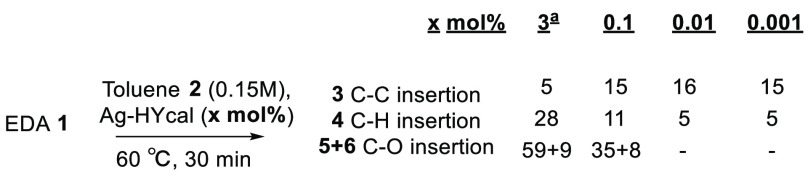
Reaction results for decreasing amounts of Ag-HYcal zeolite catalyst after 30 min of reaction time. ^a^ Results for 24 h of reaction time.

During the reaction order studies for the Ag-HYcal zeolite catalyst, we found that the amount of Ag-zeolite can be decreased very significantly, to ≤0.1 mol % in Ag, to obtain significant conversion after just 30 min of reaction. In this way, a much better selectivity for the C–C and C–H insertion products is obtained since the amount of water provided by the zeolite is minimized. Figure 10 shows this new result, which indicates that the catalytic activity of Ag in the zeolite is remarkable.

The combined HR HAADF-STEM, ^13^C CP/MAS NMR, and Raman results strongly support that Ag_*n*_-carbene complexes are formed within the zeolite, where *n* = 1–3. The selectivity toward one or another product depends on the Ag electronics, modulated by the zeolite, in such a way that electron-poor zeolites such as HY stabilize highly cationic Ag species to catalyze the insertion of the carbene in O–H bonds, while Li- to K-exchanged zeolites stabilize less cationic Ag species to catalyze the insertion of the carbene in C–H bonds. However, the stability of Ag species in less acidic zeolites seems to be lower according to reusability tests with Ag-LiNaYcal zeolite (Figure S25) and TEM analysis (Figure S26, Ag nanoparticles can be seen). Despite many reactions being investigated, these reactions can be classified in basically two groups—C–H and O–H carbene insertions—and the selectivity for one or another is the main activity of the zeolite catalysts studied here.

## Conclusions

The synthesis of ultrasmall Ag species in zeolite Y has been achieved by a simple exchange–calcination procedure to obtain cationic single- and few-atom silver clusters. This supported Ag zeolites catalyze carbene-mediated organic reactions with high yield and selectivity, including cyclohexane as a substrate, depending on the zeolite counterbalancing cation, which dictates the electronics of the Ag active site. The amount of catalyst in the reaction can be as low as ≤0.1 mol % Ag. These results showcase the high catalytic activity of Ag in organic synthesis when obtained in subnanometric form^35^ and the use of zeolites as easily tunable macroligands to generate and stabilize such ultrasmall supported metal species.^36^ Incidentally, we have found a method to deeply dry the HY zeolite by making the strongly adsorbed water react with the in situ formed carbenes.

## Supporting Information Available

Experimental section, including the synthesis of the materials and the reaction procedures, and Supporting Table S1 and Figures S1–S26 (PDF)
