# Supplementary material for: Silver-Exchanged Zeolite Y Catalyzes a Selective Insertion of Carbenes into C–H and O–H Bonds
Source: J Am Chem Soc. 2023 Nov 3;145(45):24736–45. doi: 10.1021/jacs.3c08317 (PMC10655197; doi:10.1021/jacs.3c08317)
Supplement: Supplementary file 1 — ja3c08317_si_001.pdf [file ja3c08317_si_001.pdf]

SUPPORTING INFORMATION

Silver–exchanged zeolite Y catalyzes a selective insertion of carbenes into C–H and O–H bonds

Yongkun Zheng,† Alejandro Vidal–Moya,† Juan Carlos Hernández–Garrido,†† Marta Mon*,† and Antonio Leyva–Pérez.*,†

† Instituto de Tecnología Química (UPV–CSIC), Universitat Politècnica de València–Consejo Superior de Investigaciones Científicas, Avda. de los Naranjos s/n, 46022 Valencia, Spain

†† Departamento de Ciencia de los Materiales e Ingeniería Metalúrgica y Química Inorgánica, Facultad de Ciencias, Universidad de Cádiz, Campus Universitario Puerto Real, 11510 Puerto Real, Cádiz, Spain.

Table of contents

**Experimental section**	p. S3
Materials	p. S3
Reactions	p. S3
Physical techniques	p. S3
Synchrotron experiments.	p. S4
Temperature–programmed Fourier transform infrared spec.	p. S4
Raman spectroscopy	p. S4
Solid state nuclear magnetic resonance	p. S5
Microscopy experiments	p. S5
Synthesis of the materials	p. S6
Reaction procedures	p. S7
**Supporting Tables** (Table S1)	p. S8
**Supporting Figures** (Figures S1–S26)	p. S8

Experimental section.

Materials. All reagents (≥97% purity) and solvents (≥99% purity) were purchased from commercial suppliers and used as received unless otherwise indicated.

Reactions. Glassware was dried in an oven at 175 ºC before use. Reactions were typically performed in 2.0 ml vials equipped with a magnetic stirrer and closed with a steel cap having a rubber septum part to sample out, and placed in steel heaters.

Physical techniques. Attenuated total reflection infrared spectroscopy, performed in a JASCO FT/IR–4700, was employed to record the IR spectra from 400 to 4000 cm^{-1} of the different solid catalysts. Absorption spectra were recorded on a spectrophotometer under diffuse reflectance mode. Fluorescence spectra were obtained with a LP S–220B (Photon Technology International) equipped with 75 W Xe lamp. Thermogravimetric analyses were performed on 0.5–0.8 mm pelletized samples under a dry N_2 atmosphere with a thermobalance operating at a heating rate of 10 ºC min^{-1} from 25 to 450 °C. N_2 adsorption–desorption isotherms were performed at 77 K on sieved zeolites after outgassing for 16 h under vacuum with a Micromeritics ASAP2020 instrument. Gas chromatographic analyses were performed in an instrument (Shimadzu GC–2025) equipped with a 25 m capillary column of 50 %–phenyl– 50 %–dimethylpolysiloxane. *N*–dodecane was used as an external standard. GC/MS analyses were performed on a spectrometer equipped with the same column as the GC (Agilent GC 6890 N coupled with Agilent MS–5973) and operated under the same conditions. ^{1}H, ^{13}C and DEPT nuclear magnetic resonance (NMR) spectra were recorded at room temperature on a 400 MHz spectrometer (Bruker Ascend 400) using the appropriate solvent and containing TMS as an internal standard. All the organic products obtained were characterised by GC–MS, ^{1}H–, ^{13}C–NMR and DEPT, and IR. The characterisation given in the literature

was used for comparison.The metal content of the solids was determined by inductively coupled plasma–atomic emission spectroscopy (ICP–AES) after disaggregation of the solid in hydrofluoric acid and later dilution. X–ray photoelectron spectroscopy (XPS) measurements of the zeolites were performed after sticking, without sieving, the zeolite onto a molybdenum plate with scotch tape film, followed by air drying. Measurements were performed on a SPECS spectrometer equipped with a Phoibos 150 MCD–9 analyzer using non–monochromatic Mg KR (1253.6 eV) X–ray source working at 50 W. As an internal reference for the peak positions in the XPS spectra, the C1s peak has been set at 284.5 eV. X–Ray diffraction spectra of the different catalysts were recorded in a CubiX PRO (PAN Analytical) spectrometer, with a Cu K(α) radiation source, 1.5406 Å wavelength.

Synchrotron experiments. X–ray absorption experiments, at the Ag K–edge, were performed at ALBA synchrotron (Cerdanyolla del Vallès, Spain). The white beam was monochromatized using a Si (311) double crystal cooled by liquid nitrogen; harmonic rejection was performed using Rh–coated silicon mirrors. The spectra were collected in transmission mode. Reference patterns (e.g. metal foil) were measured simultaneously between I1 and I2 and used for spectra alignment.

Diffuse reflection infrared Fourier transform spectroscopy (DRIFTS) of adsorbed CO. DRIFTS using CO as a probe molecule was used to evaluate electronic properties of Ag – HY. The experiments have been carried out in a homemade IR cell able to work in the high and low (77 K) temperature range. Prior to CO adsorption experiments, the sample was activated at 473 K under vacuum (10^{-6} mbar) for 2 h. CO adsorption experiments were performed at 77 K in the 0.2–20 mbar range. Spectra were recorded once complete coverage of CO at the specified CO partial pressure was achieved

Raman spectroscopy. Raman spectra were recorded at RT with a 514 nm laser excitation on a Renishaw Raman Spectrometer ("in via") equipped with a CCD detector. The laser power on the sample was 25 mW and a total of 20 acquisitions were taken for each spectrum. The experiments were carried out using a commercial Linkam FTIR600 catalytic cell. For the in situ study the sample has been activated in a flow of N_2 at 200 ºC for 1.5 h. After activation the sample has been cooled down and EDA **1** has been adsorbed by flushing N_2 at room temperature through a saturator containing **1**. Spectra have been acquired with time until a total of 1 h.

In–situ magic angle spinning–solid state nuclear magnetic resonance (MAS ss–NMR) experiment. 200 mg of zeolite (1 wt%) were introduced into a glass insert and degassed at 200 °C under vacuum during 18 h. Then, EDA **1** was introduced with a nitrogen stream while the system was still under vacuum, and the glass insert was sealed immersed into liquid nitrogen. ^{13}C solid–state NMR spectra were recorded at room temperature with a Bruker AVIII HD 400 WB spectrometer. The glass insert was fitted into 7 mm rotors and were spun at 5 kHz in a Bruker BL7 probe. ^{13}C CP/MAS NMR spectra were recorded with proton decoupling, with ^{1}H 90° pulse length of 5 μs, and a recycle delay of 3 s.

Microscopy experiments. Samples for electron microscopy studies were prepared by dropping the suspension of the solid sample in DCM directly onto holey–carbon–coated copper grids. For Ag–containing base zeolite, powder sample were embedded in an epoxy resin and sliced at a thickness of less than 100 nm with an ultramicrotome. They were then deposited on holey carbon copper grid before TEM observation. HR HAADF–STEM and STEM–iDPC studies were performed on a double–aberration–corrected, monochromated, FEI Titan^3 Themis 60–300 microscope working at 300 kV. 2048 × 2048

HAADF–iDPC image pairs were recorded simultaneously using a convergence angle of 18.6 mrad and a camera length of 230 mm and collection angles of 25–153 mrad and 115 mm with 42–198 mrad collection angle. This configuration allowed us to optimize the collection of the signals on the HAADF and FEI DF4 detectors. To limit the damage by the electron beam, a fast image–recording protocol was used by combining a beam current of 5–3 pA, a 0.63 μs dwell time (corresponding to dose rates of 95–200 $e^{-}/Å^{2}$). iDPC imaging provides atomically resolved images by using a four–segment detector, and this technique allows imaging light elements, such as O, in the presence of heavier ones (Si, Z = 14) under very low–electron–dose conditions, a key aspect in the atomic–scale structural analysis of zeolites, which are very sensitive to electron beams. In particular, to determine the spatial distribution of the silver species within the zeolite framework, a specific methodology for the digital analysis of the experimental images has been applied and coded in a home–made MATLAB script. First, to improve the signal–to–noise, the HR HAADF–STEM images were denoised by combining the Anscombe transform and undecimated wavelet transforms. Then, a fully automated segmentation of image contrasts by clustering techniques (*k*–means method) was applied to recognize and classify the metallic entities, which is a requirement to guarantee statistically meaningful and unbiased results. To support the *k*–means clustering analysis and interpretation of the details of the experimental images, HR HAADF–STEM image simulation was carried out using TEMSIM software. The complex structural models used as input in these simulations were built using the Rhodius software developed at University of Cádiz.

Synthesis of the materials.

General procedure for the preparation of basic zeolites and Ag^{+} incorporation. Commercially available Y zeolite, in Na^{+}–form, were treated with a 1M aqueous solution

of Li^+, K^+ or Cs^+ acetate, to give the corresponding Li^+–, K^+– and Cs^+–zeolites (LiNaY, KNaY and CsNaY). The resulting solids were further cationically exchanged with an aqueous solution of $AgNO_3$. In this way, Ag is homogenously distributed along the zeolite channels and cavities. Finally, zeolites Ag–LiNaY, Ag–NaY and Ag–KNaY were calcined at 450 °C.

Reaction procedures.

General reaction procedure: The corresponding Ag solid catalyst (3 mol% Ag) and the aromatic substrate (0.8 mL) were introduced in a glass vial equipped with a magnetic stirrer, ethyl diazoacetate (EDA **1**, 0.1 mmol) was added, and the vial was sealed. Then, the vial was placed in a magnetically stirred pre–heated oil bath at 60 ºC overnight. After the reaction is complete, filtration is carried out to separate the solid catalyst. The reaction mixture was analyzed by GC and GC–MS.

Hot filtration test: Following the general reaction procedure, two parallel reactions were carried out and one of them was rapidly filtrated at the reaction temperature (60 ºC) after 20 min reaction time (~30% conversion). Then, the kinetic profiles for both the solid–containing reaction and the filtrates were assessed and compared.

Reuses: Following the general reaction procedure, the solid catalyst was separated by centrifugation at 4000 r.p.m. during 5 min, washed with dichloromethane (1 mL) for three times, separated again and dried. Fresh reactants were placed for a new reaction.

Supporting Tables

Table S1. Inductively coupled plasma atomic emission spectroscopy (ICP–AES) results for the different Ag–zeolites.

Entry	Zeolite	Ag%	Na%	Li/K/Cs%
1	Ag–HYCal	1.08	–	–
2	Ag–LiNaYCal	0.94	5.98	1.22
3	Ag–NaYCal	0.77	6.30	-
4	Ag–KNaYCal	1.33	2.38	7.13
5	Ag–CsNaY	1.13	2.49	3.81

Supporting Figures

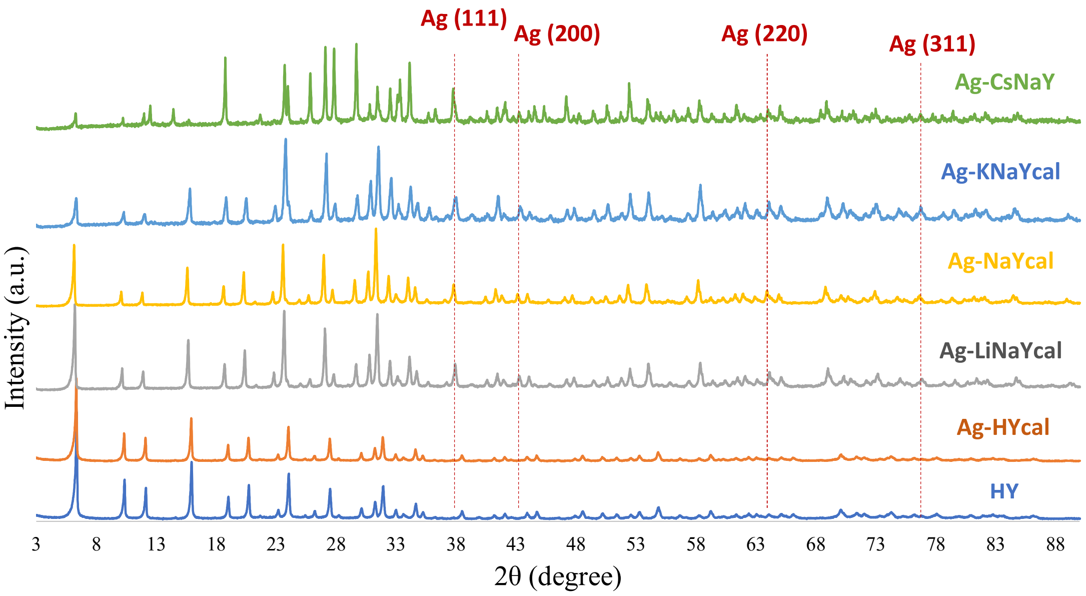

Figure S1. Powder X–ray diffractograms (XRD) of the different Ag–zeolites, indicating the formation or not of different crystallite planes of Ag nanoparticles.

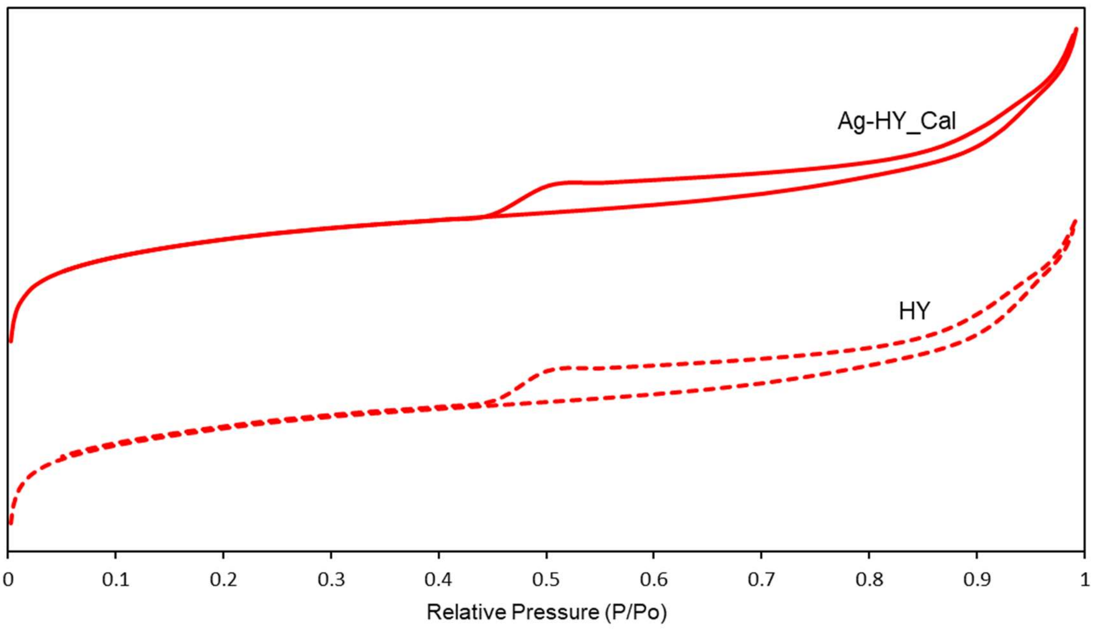

Figure S2. Brunauer–Emmett–Teller surface area plots (BET) of HY (top) and Ag–HYcal zeolite (bottom). Ag–HYcal zeolite: BET Surface Area: 708.76 m^2/g, t–Plot micropore volume: 0.312 cm^3/g; HY zeolite: BET Surface Area: 723.38 m^2/g, t–Plot micropore volume: 0.317 cm^3/g.

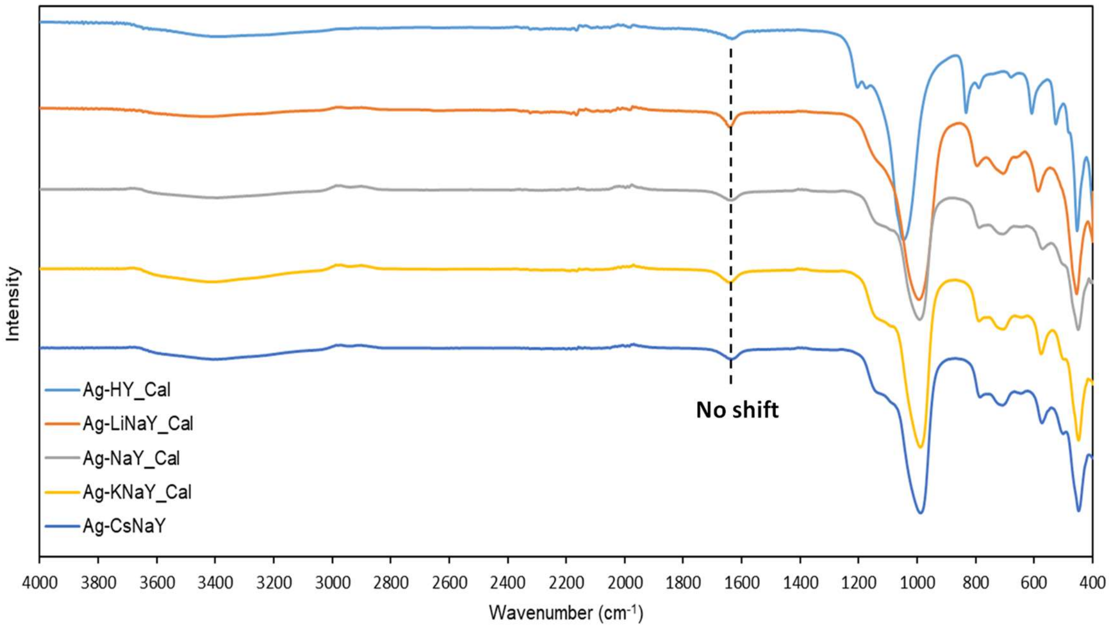

Figure S3. Fourier–transformed infrared (FT–IR) spectroscopy of the different Ag–zeolites. The stretching band of adsorbed water, external to the framework, remains unvaried as indicated (~1630 cm^{-1}).

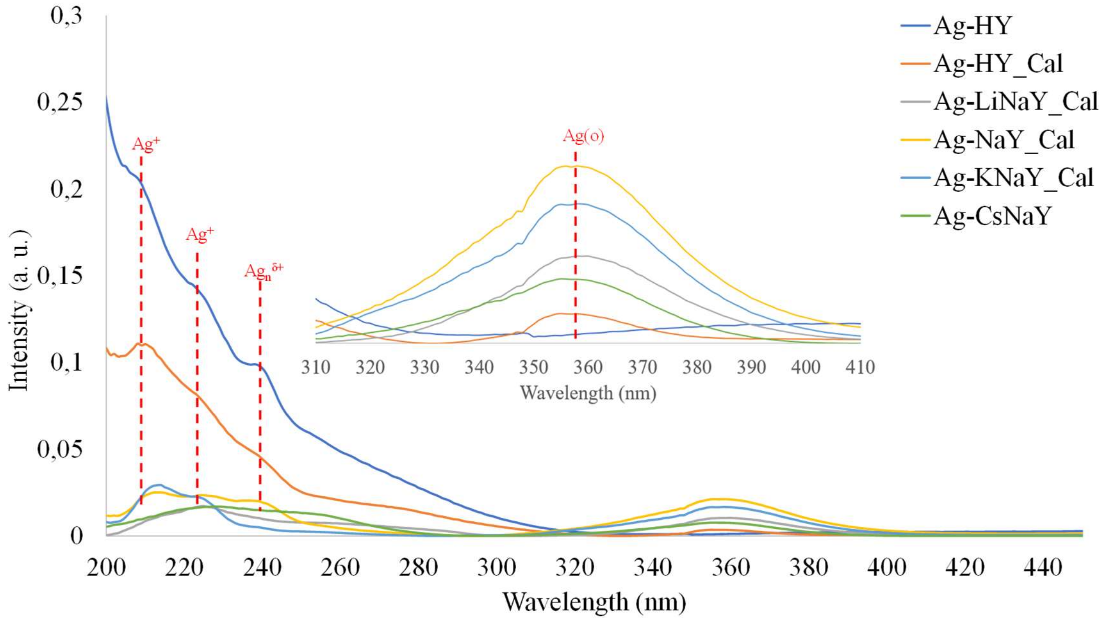

Figure S4. Diffuse reflectance UV–vis spectrophotometry (DR–UV–vis) of the different Ag–zeolites. The area around 360 nm is magnified, and the most prominent bands together with their plausible assignations are indicated.

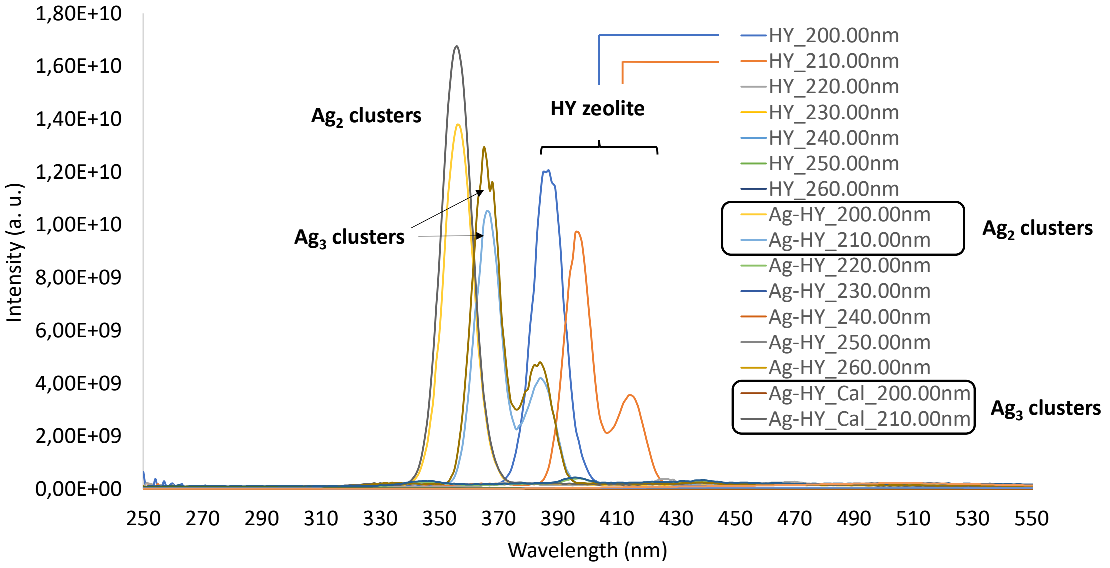

Figure S5. UV–vis emission spectrophotometry (fluorescence UV–vis) of HY and Ag–HY zeolites at different excitation wavelengths.

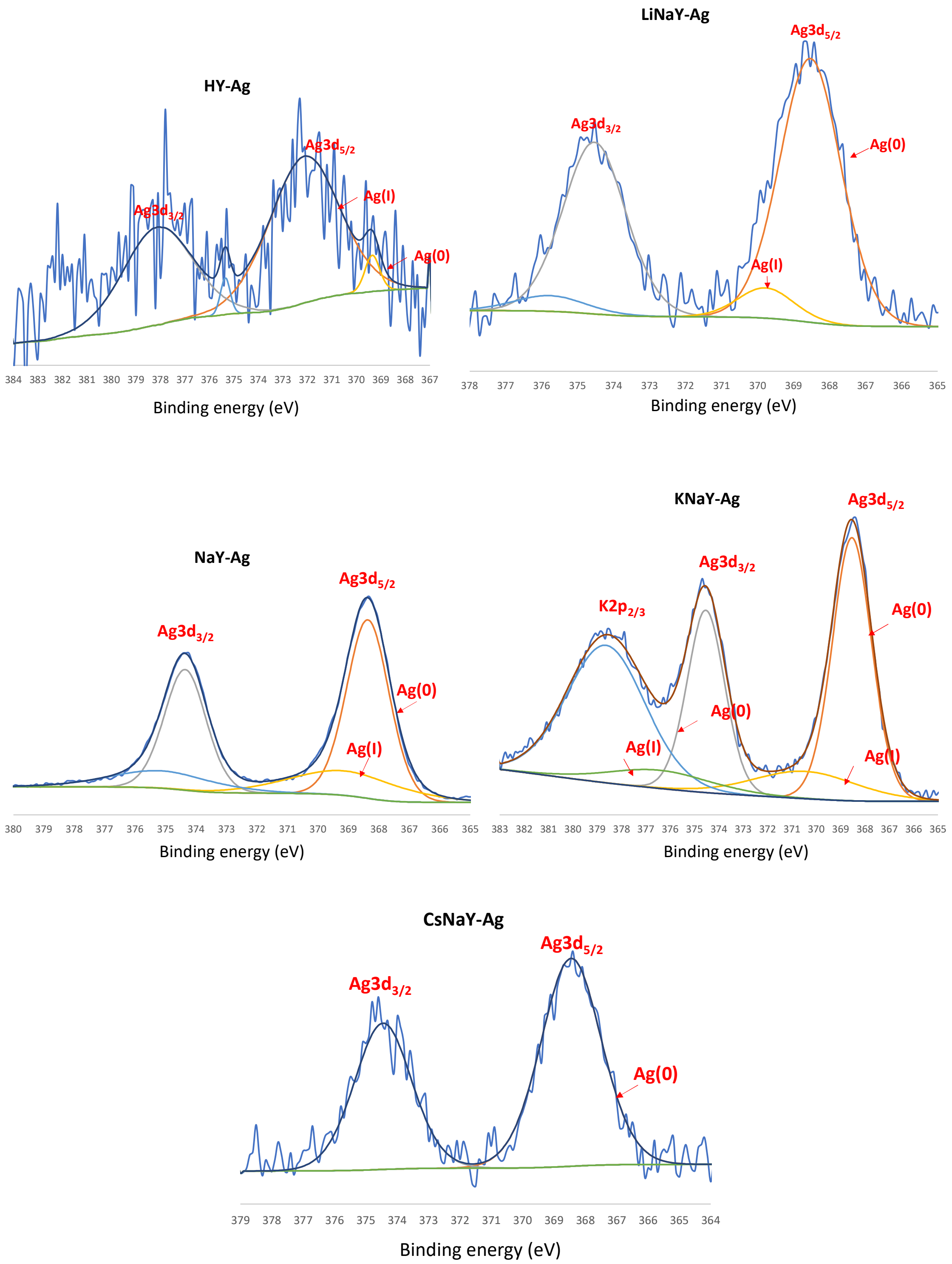

Figure S6. Deconvoluted X–ray photoelectron spectra (XPS) for $Ag3d_{5/2}$ for the different cation–exchanged Ag–zeolites, indicating the amount of cationic Ag (Ag^{+}) and reduced Ag (Ag^{0}).

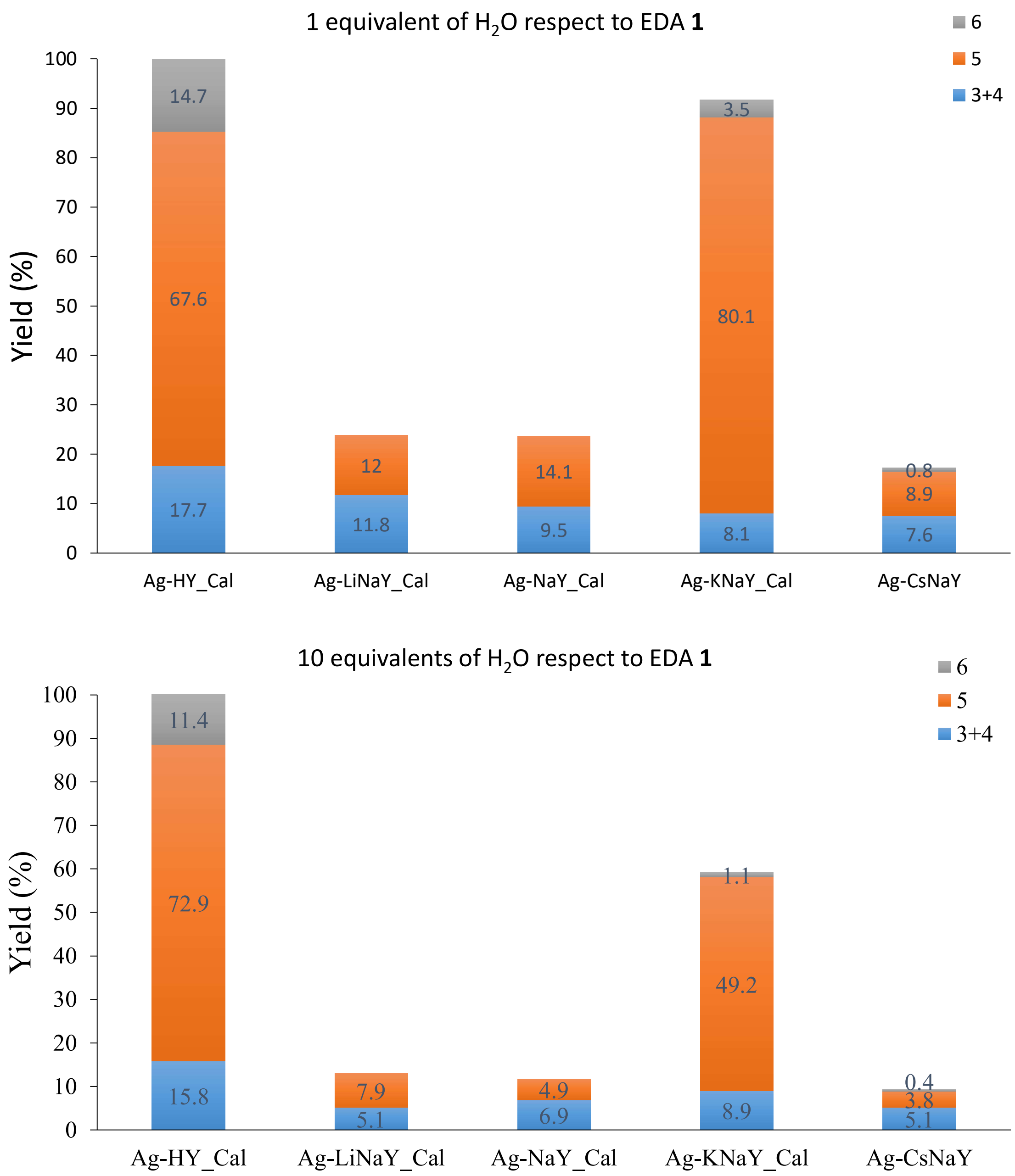

Figure S7. Catalytic results for the reaction of ethyldiazoacetate (EDA) **1** in toluene solvent (0.15M) with either 1 (top) or 10 equivalents of added water (bottom) and different Ag–zeolite catalysts (3 mol% Ag), at 60 ºC for 24 h. Complete conversion of **1**. Error bars account for a 5% uncertainty. For the structure of products **3–6** see the main text.

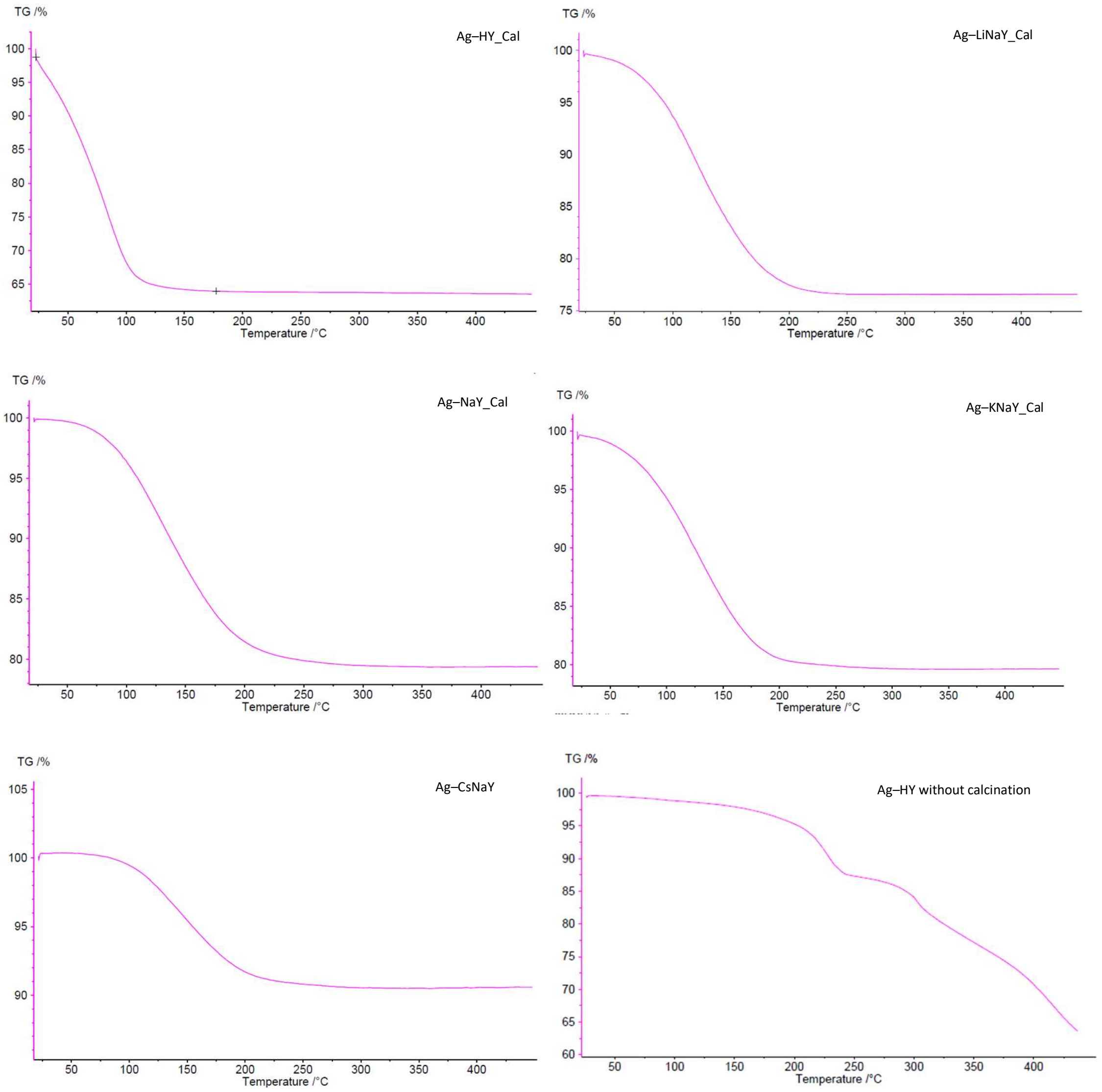

Figure S8. Thermogravimetric analysis (TG) of the different cation–exchanged zeolites after calcination (except for CsNaY). The last spectrum corresponds to Ag–HY without calcination.

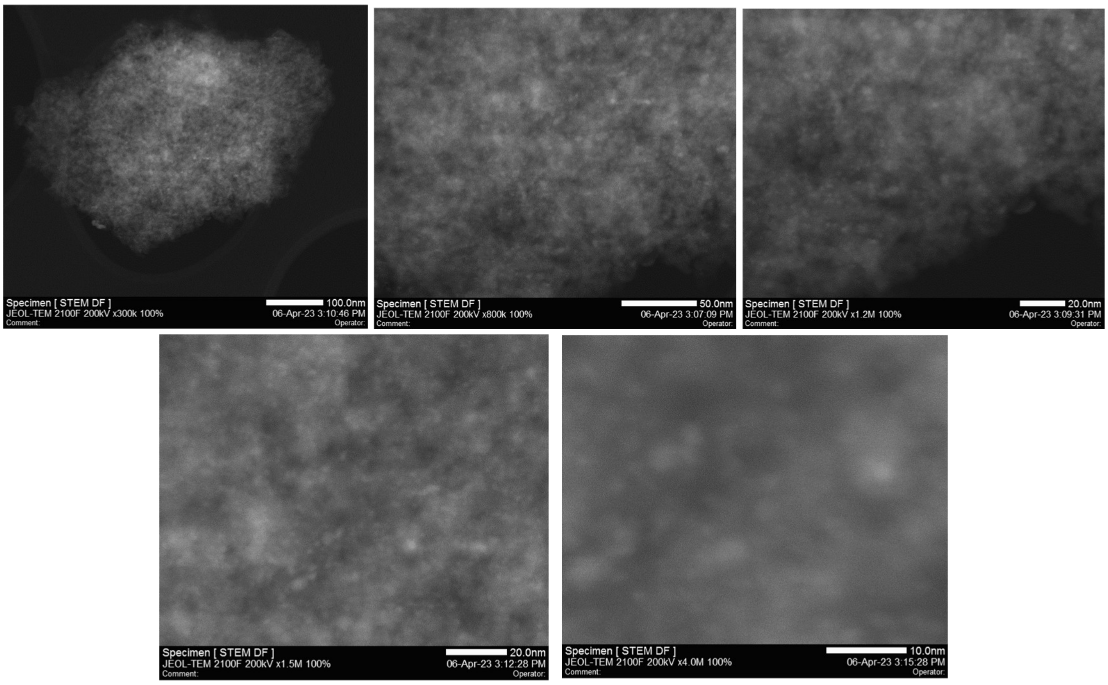

Figure S9. High resolution transmission electron microscopy (HR–TEM) images of $Ag–Al_2O_3$ at increasing magnifications. The average nanoparticle size is ~2 nm.

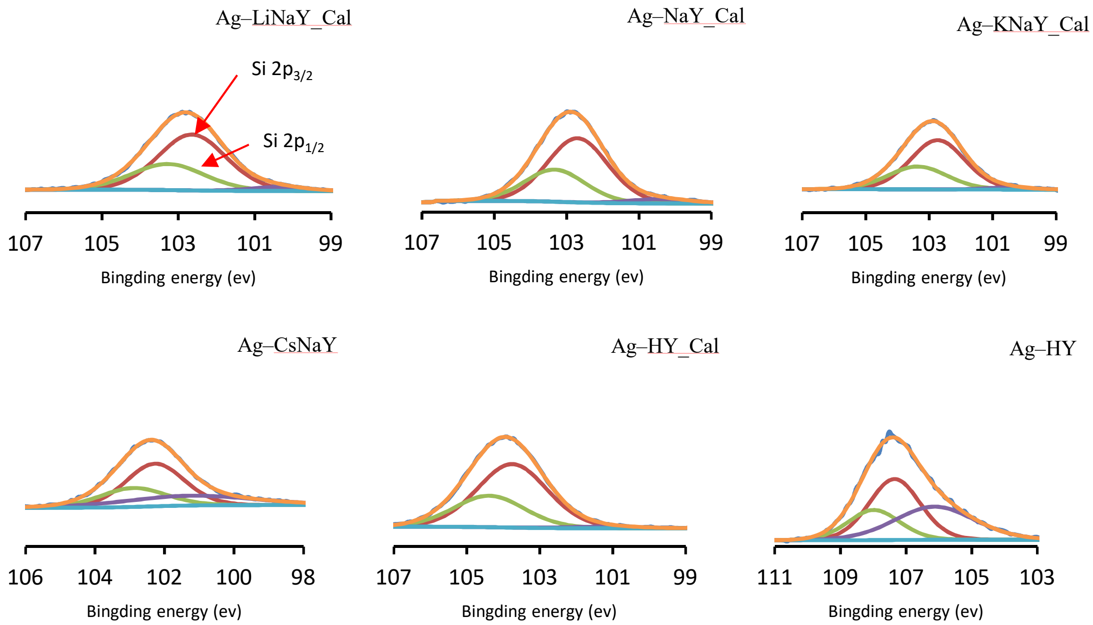

Figure S10. Deconvoluted X–ray photoelectron spectra (XPS) for $Si2p_{3/2 \text{ or } 1/2}$ in the different Ag–zeolites.

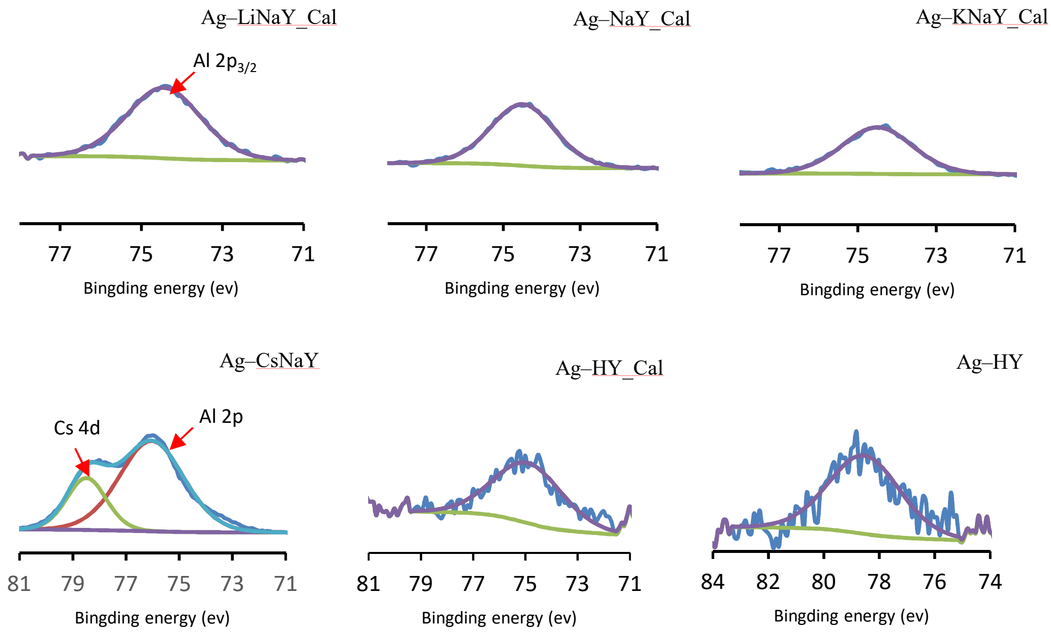

Figure S11. X–ray photoelectron spectra (XPS) for Al2$p_{3/2}$ in the different Ag–zeolites.

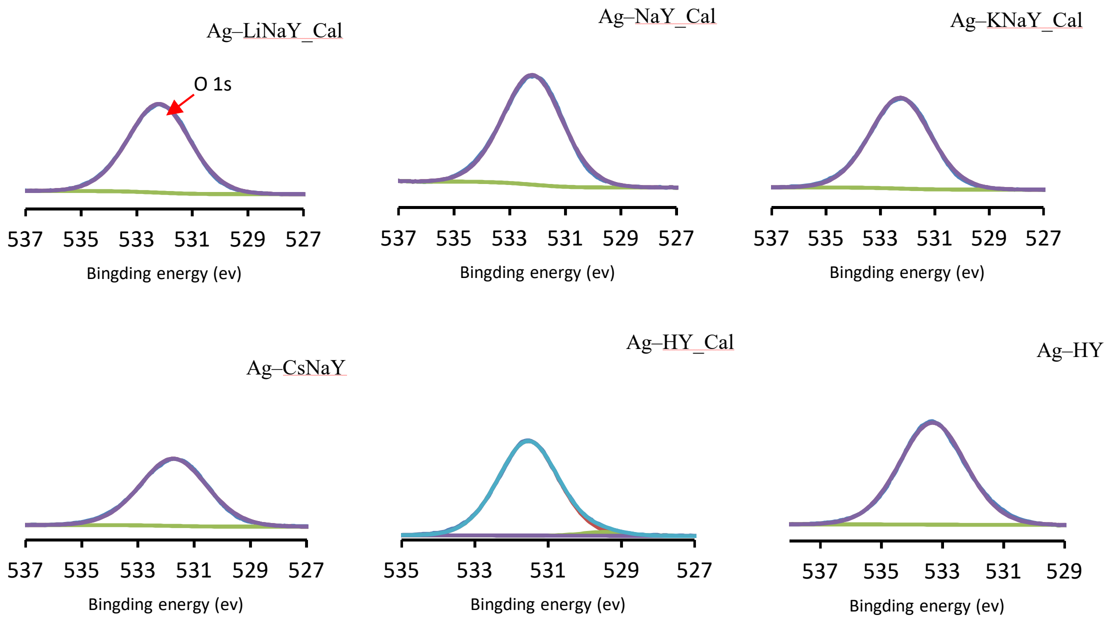

Figure S12. X–ray photoelectron spectra (XPS) for O1s in the different Ag–zeolites.

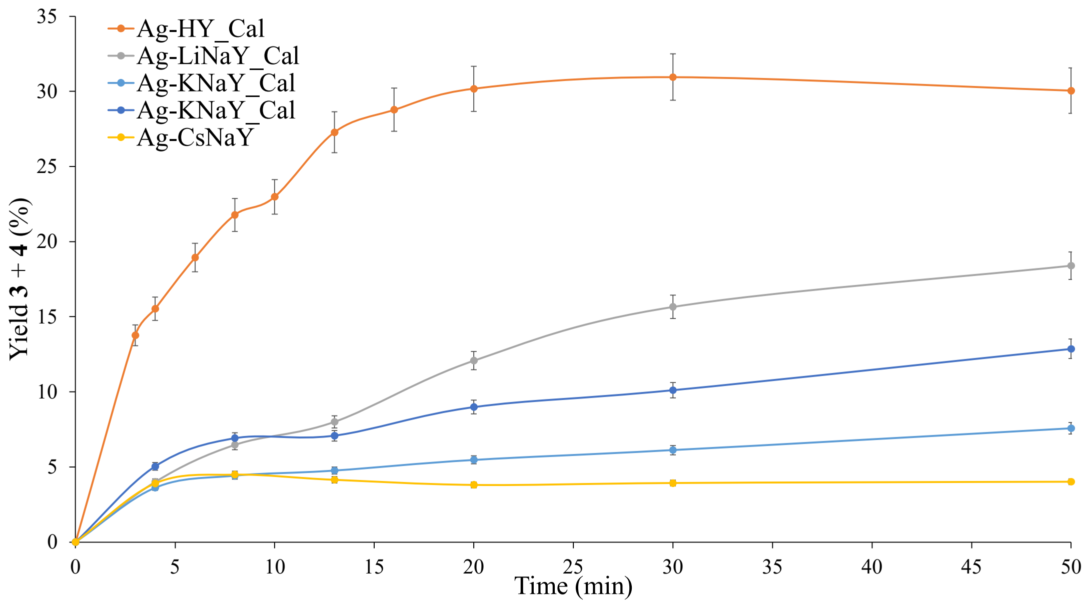

Figure S13. Kinetic plot for the first 50 min reaction time of the reaction of ethyldiazoacetate (EDA) **1** in toluene solvent (0.15M) at 60 ºC with different Ag–zeolite catalysts. The formation rate of the O–H insertion products **5** and **6** is omitted for clarity. Error bars account for a 5% uncertainty.

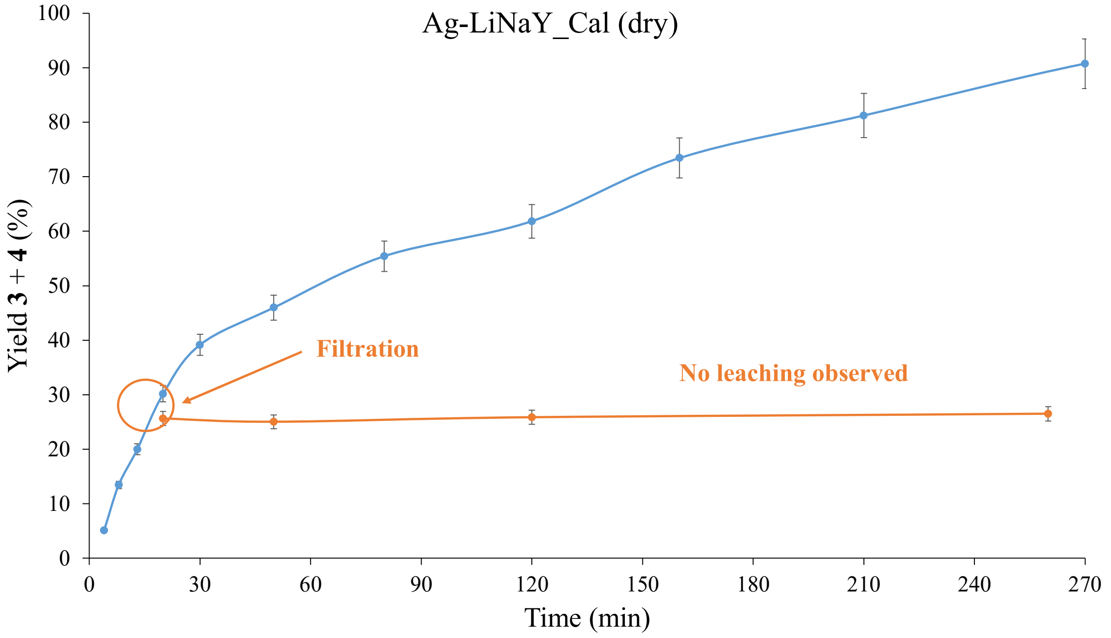

Figure S14. Hot filtration test for the Ag–LiNaYcal zeolite catalyst during the reaction of ethyldiazoacetate (EDA) **1** in toluene solvent (0.15M) at 60 ºC. Error bars account for a 5% uncertainty.

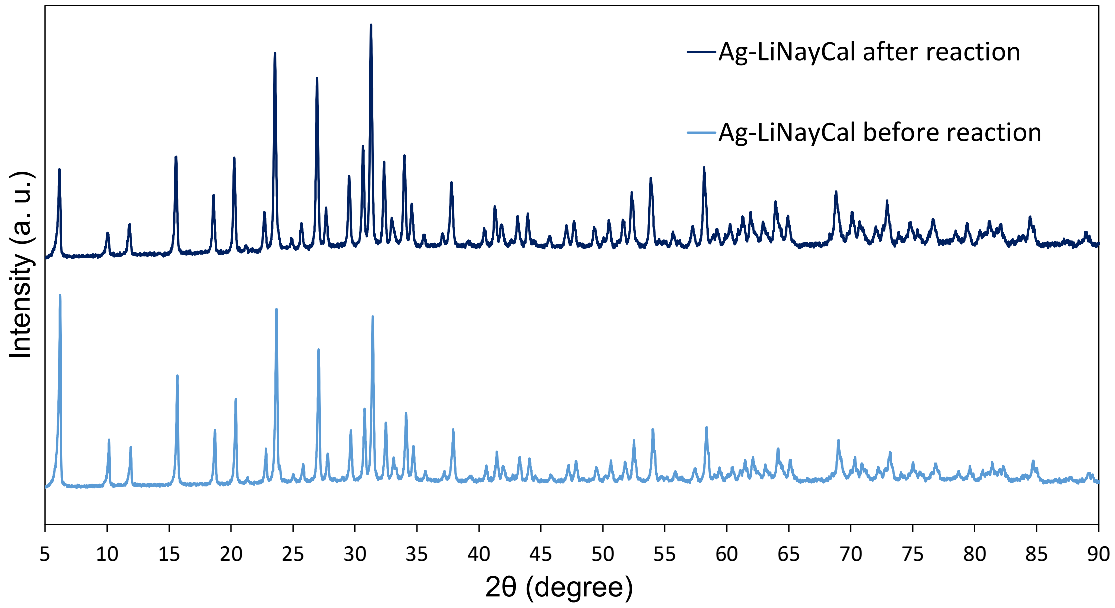

Figure S15. Comparison of the X–ray diffractograms (XRD) of the Ag–LiNaYcal zeolite catalyst before and after the reaction of ethyldiazoacetate (EDA) **1** in toluene solvent (0.15M) at 60 ºC for 24 h.

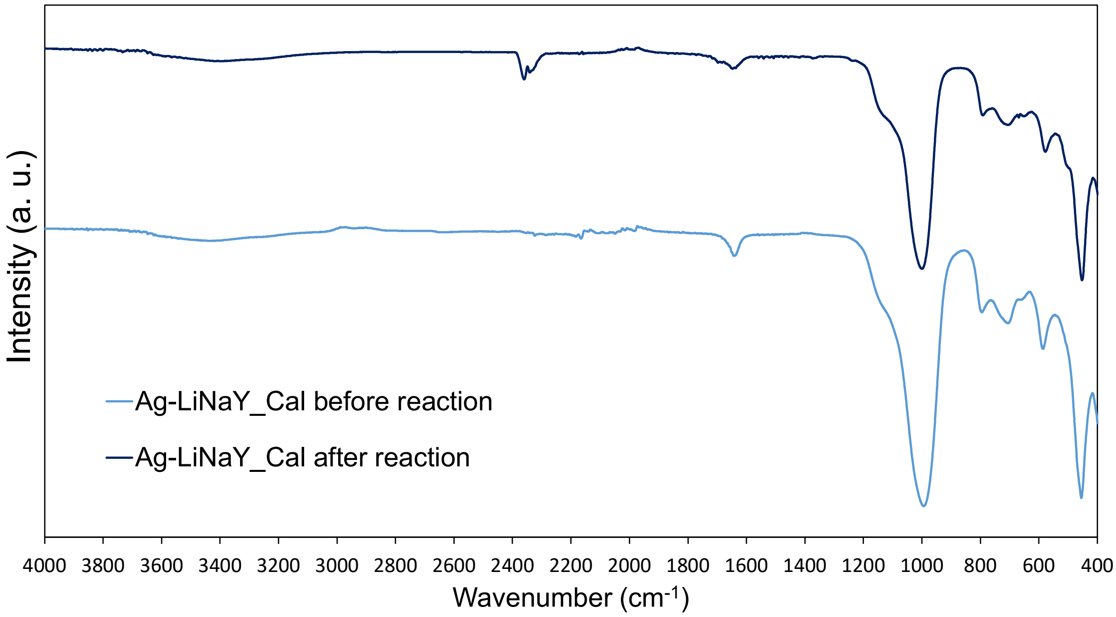

Figure S16. Comparison of the Fourier–transformed infrared (FT–IR) spectra of the Ag–LiNaYcal zeolite catalyst before and after the reaction of ethyldiazoacetate (EDA) **1** in toluene solvent (0.15M) at 60 ºC for 24 h. The peak at 2330 cm^{-1} corresponds to residual CO_2 in the FT–IR chamber.

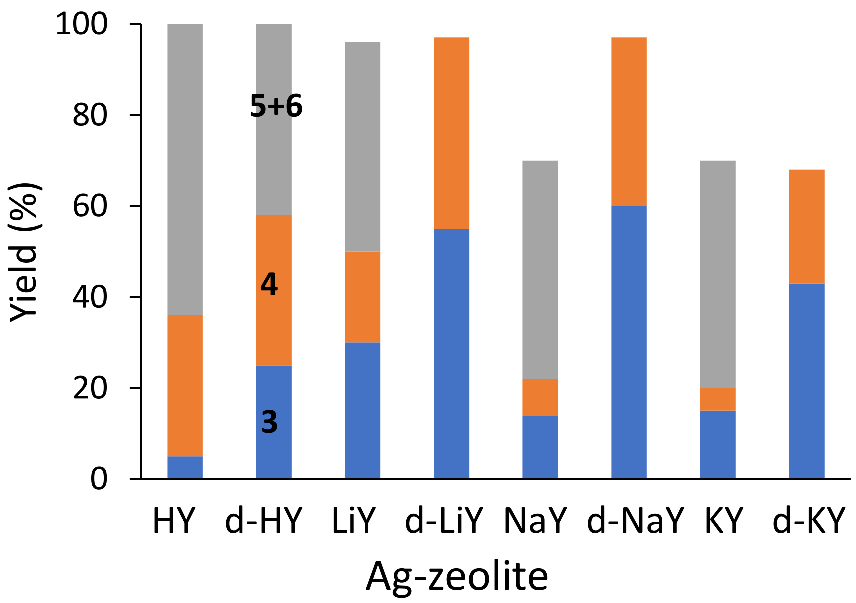

Figure S17. Comparison of the catalytic results for the different Ag zeolites before or after dehydration of the zeolite (denoted as a "d-").

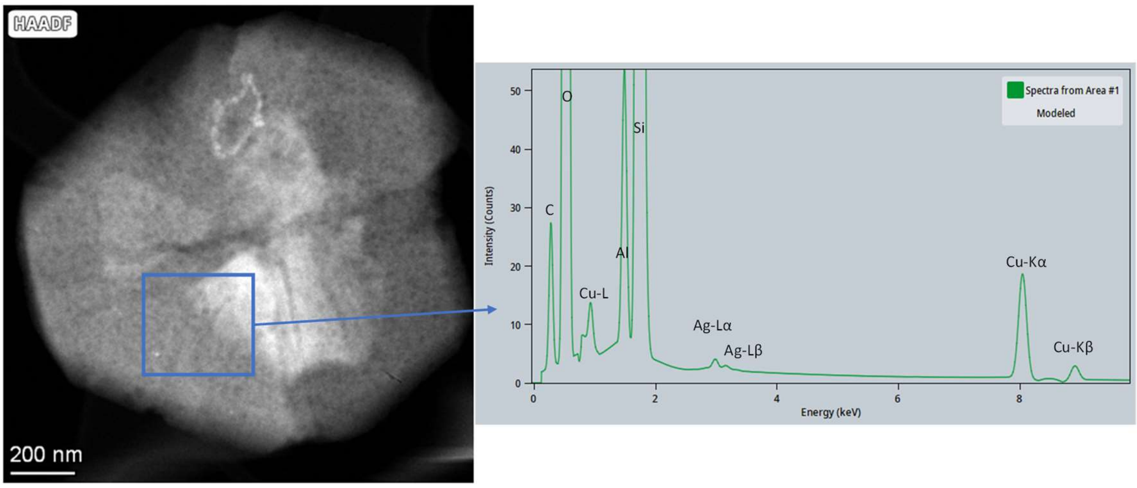

Figure S18. High–angle annular dark–field scanning transmission electron microscopy (HAADF–STEM) image of a Ag–LiNaYcal crystallite (left) and the corresponding energy–dispersive X–ray spectroscopy (EDX) elemental analysis from the squared area, including quantification results. Cu signals corresponds to the Cu–carbon grid employed.

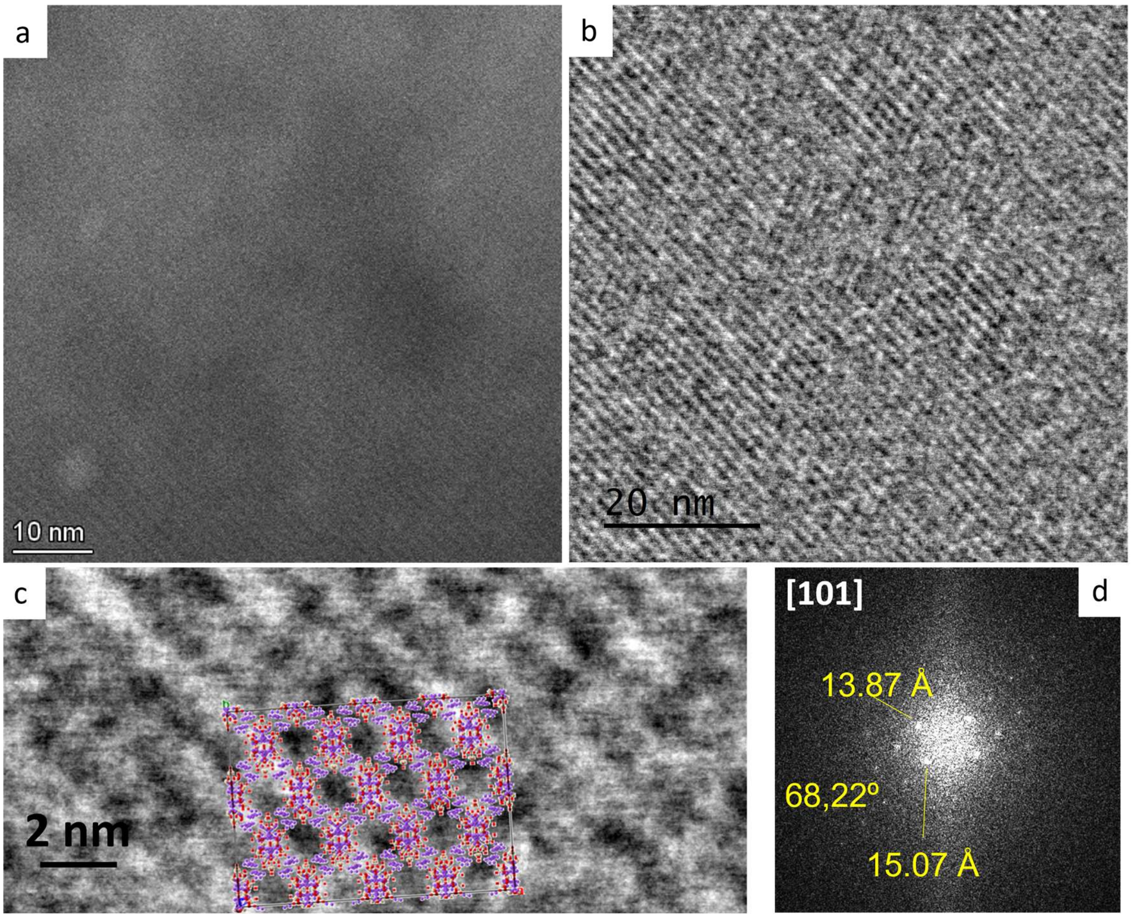

Figure S19. (a) Aberration corrected high–angle annular dark–field scanning transmission electron microscopy (AC HAADF–STEM) image of a Ag–LiNaYcal crystallite and (b) the corresponding integrated differential phase contrast (iDPC) image. The visualization of the Ag species is clearly shown by the HAADF mode thanks to contrast, which is roughly proportional to the square atomic number Z^2, whereas for the iDPC mode, the resulting contrast is roughly proportional to the atomic number Z, which drastically improves the detectability of light elements among heavy elements. (c) Detailed visualization of the crystallographic structure of the LiNaYcal zeolite, with modelled crystal structure superimposed, viewed along the [101] zone axis. (d) FFT obtained.

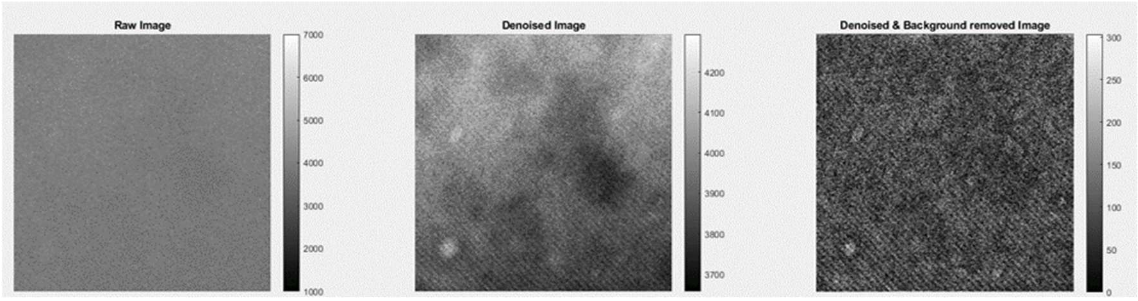

Figure S20. AC HAADF–STEM image of the Ag–LiNaYcal sample acquired at 1.4 million magnifications (left) and the corresponding images after de–noising (center) using undecimated wavelet transforms (UWT) and background subtraction (right).

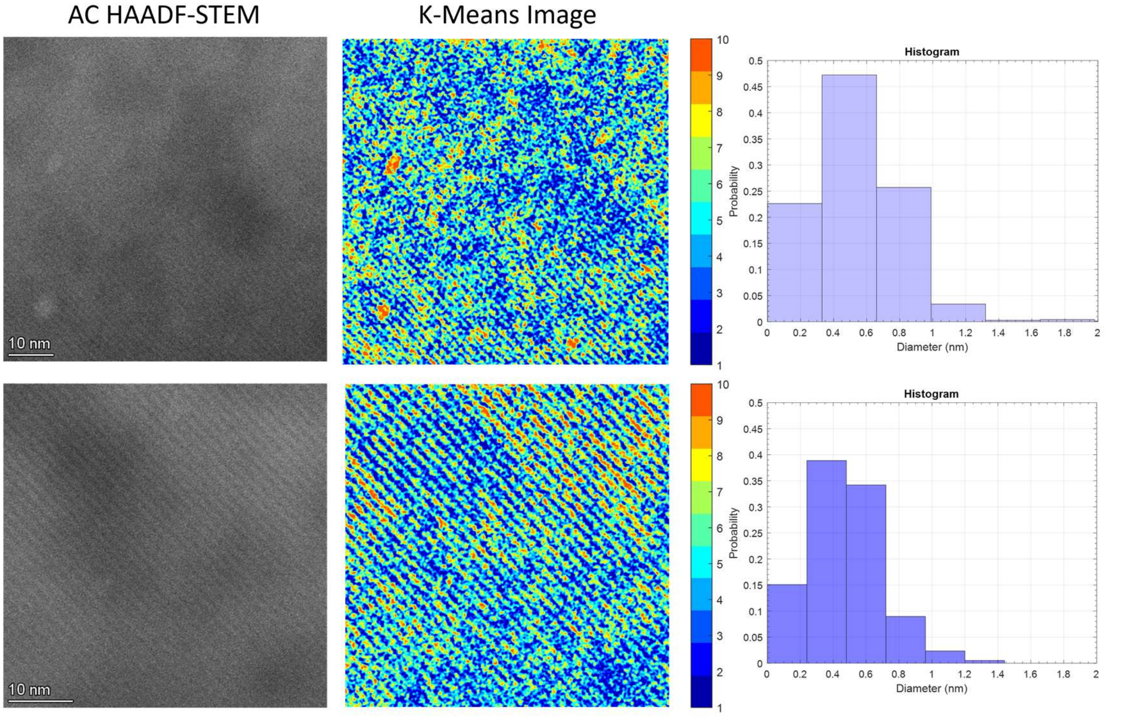

Figure S21. (Left row) Experimental AC HAADF–STEM images of Ag–LiNaYcal sample. (Middle row) K–Means clustering analysis of the experimental images after denoising and background subtraction. The K–means clusters analysis of the experimental images, shown that the pixels corresponding to clusters from 8 to 10 atoms can appropriately account for the projected size of the Ag clusters. The segmentation of the clustered images using this threshold allowed to estimate the Ag clusters size as the diameter of the circle whose area equals that of the segmented particle (equivalent circle diameter). Please, note that the word cluster in images refers to the image clustering analysis and not to Ag clusters. (Right row) The size distribution histograms, indicate that the majority of these Ag clusters fall in the size range 0.2 – 0.6 nm, in good agreement with the expected values for isolated atoms and ultrasmall Ag clusters. The remaining part of the distribution, with size in the range 0.7–2 nm, should correspond either to Ag clusters with a larger number of atoms or, instead, to the superposition of neighboring Ag clusters in the same area.

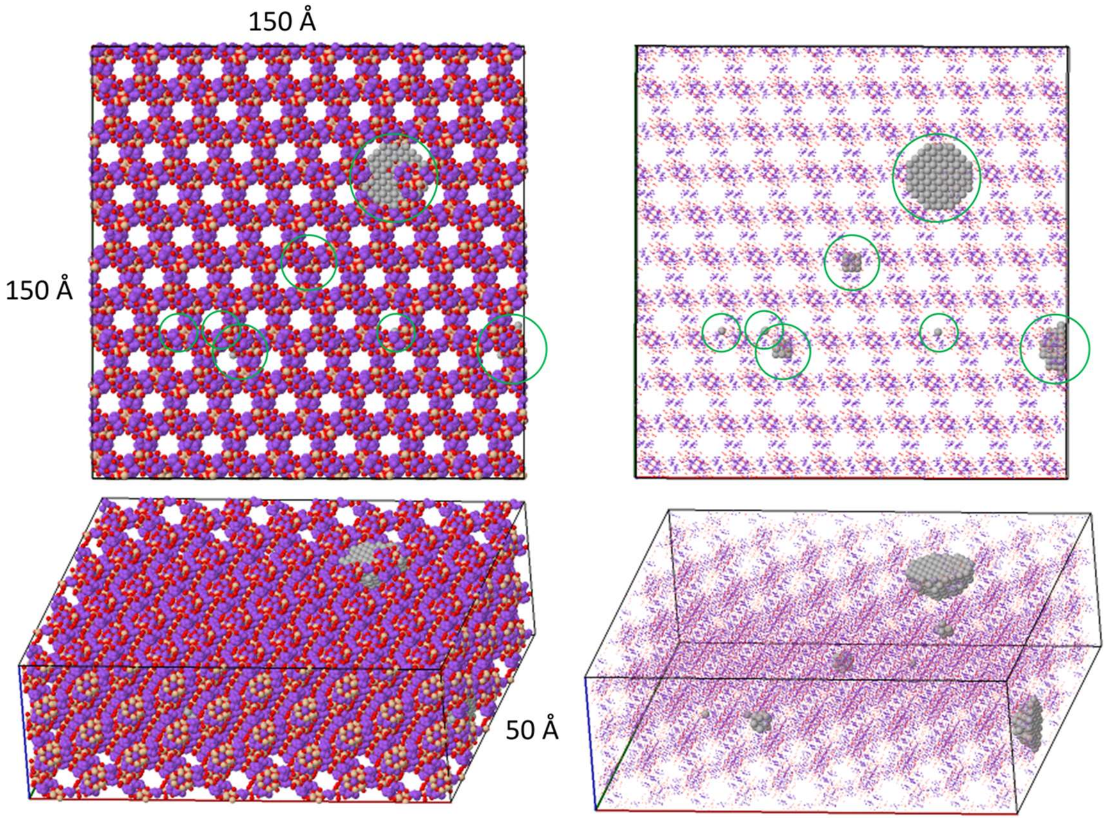

Figure S22. 150Å × 150Å × 50Å supercell in [101] zone axis including Ag species ranging from single atom to nanometric clusters. By adjusting the orientation of the model, we were able to simulate the images obtained from the experimental studies. Just for a better visualization purpose of the Ag species in this figure, the sizes of the atoms in the right zeolite models were reduced.

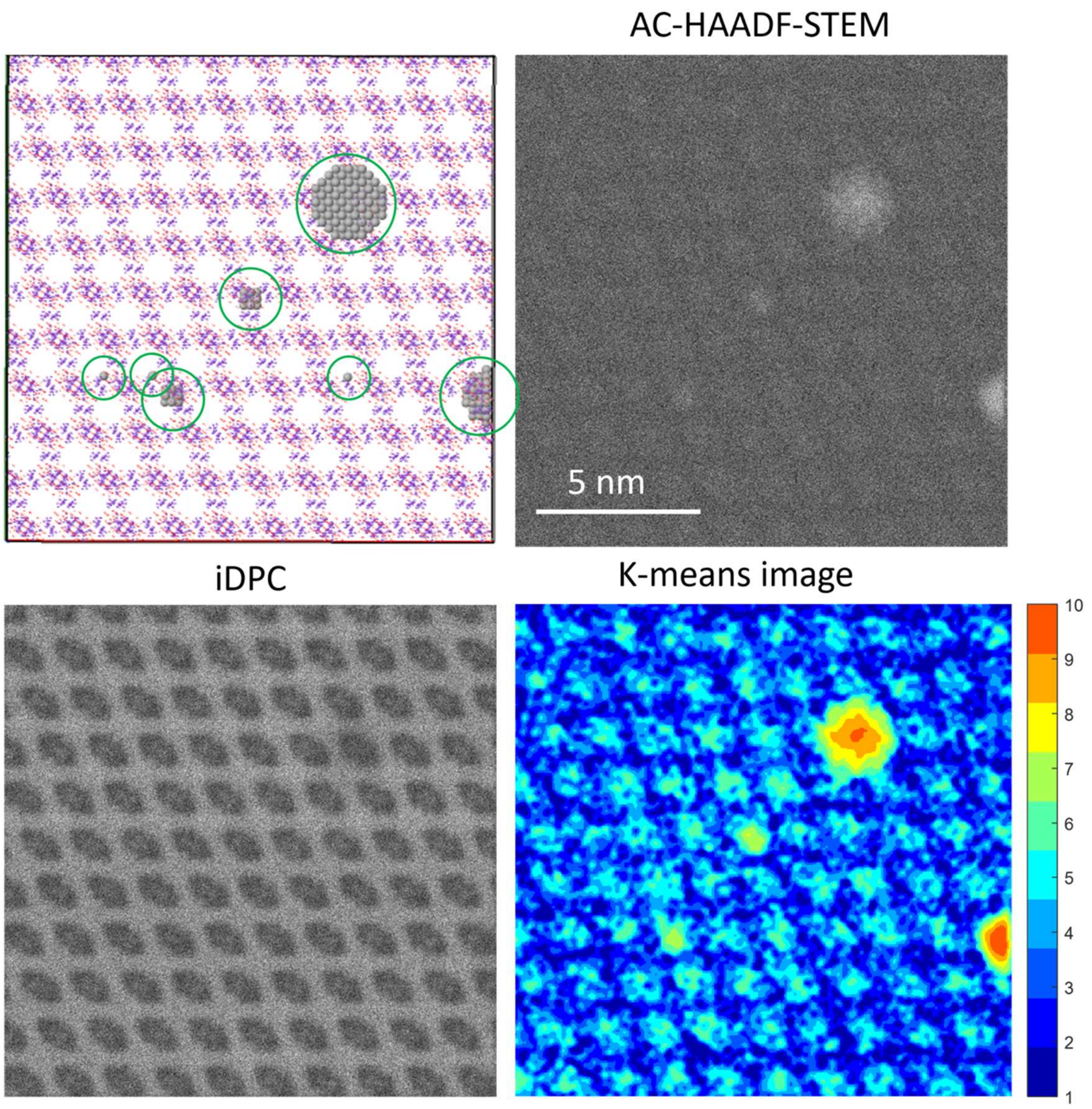

Figure S23. Image simulation details of different types of Ag single atom, Ag clusters and Ag nanoparticles in the Ag-HY zeolite along [101] direction. To approach as close as possible to the experimental imaging conditions, a mixture of Poisson and white Gaussian noise was added to the simulated images. **Top row:** Structural model implementing Ag species and simulated High Resolution HAADF–STEM simulated image after the addition of Poisson and white Gaussian noise. **Bottom row:** Simulated iDPC image illustrating the only light elements are detected using this technique (i.e. light elements). HR HAADF–STEM image. UWT de-noised image, background subtracted and de-noised image, followed by K-means clustering analysis. For Ag single atoms, the intensity is very low barely distinguished from the zeolite structure; for Ag small cluster, the intensity is a little bit higher, in some cases even down to the contrast level of the zeolite framework; in the case of Ag nanoparticles, the contrast provided is slightly higher than the zeolite framework, a situation that mimic the situation observed in the experimental images.

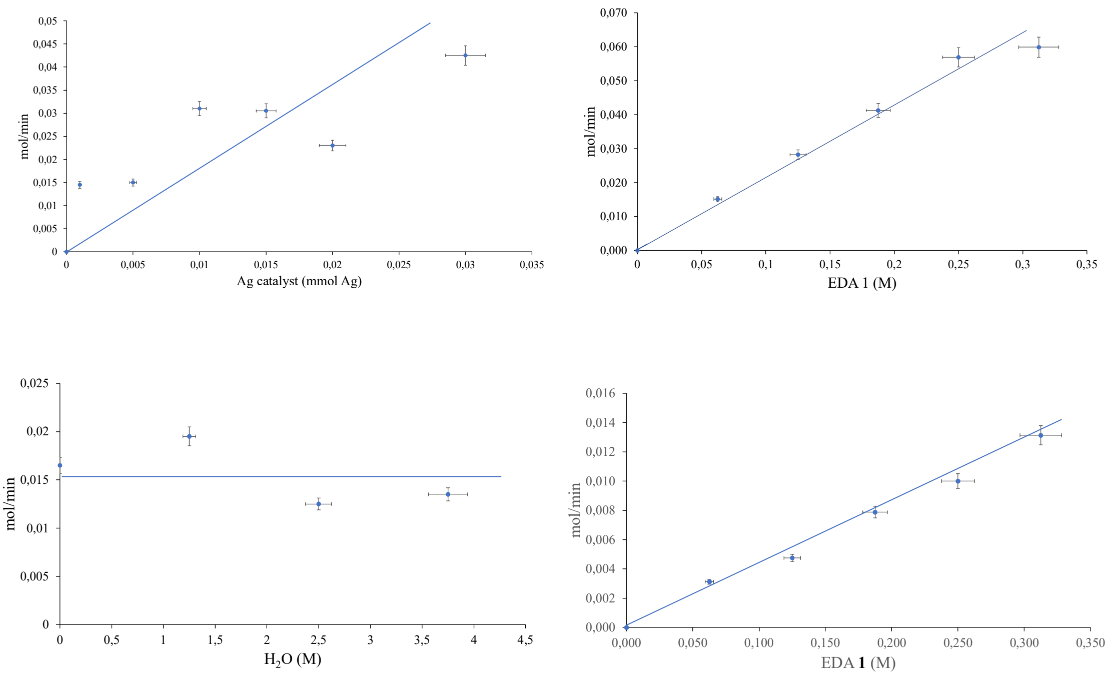

Figure S24. From top left to bottom right: Initial rates vs reagent concentration plots for the Ag-HYcal zeolite catalyst, EDA **1** and water, during the carbene insertion reaction, and the relationship with EDA **1** concentration for Ag- LiNaYcal zeolite catalyst. Error bars account for a 5% uncertainty.

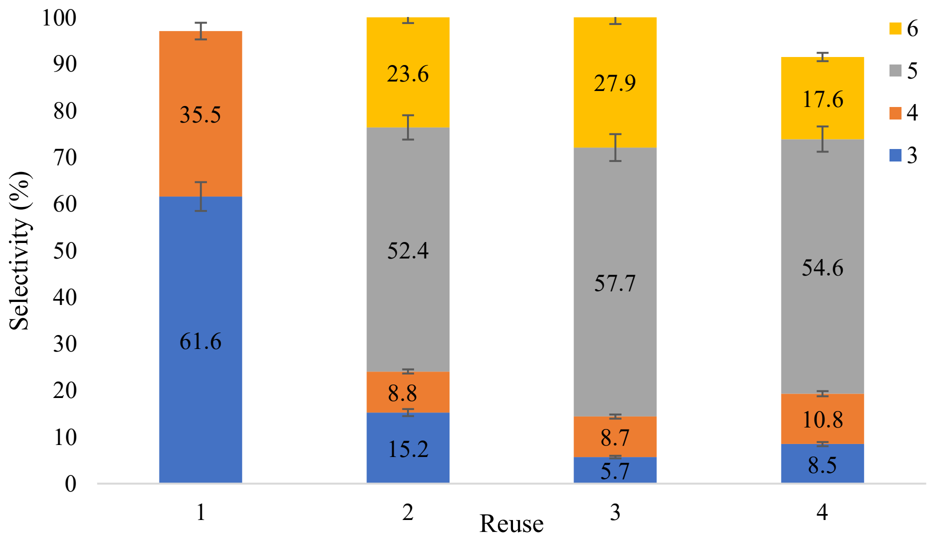

Figure S25. Reuses of Ag–LiNaY as a catalyst (3 mol% Ag) for the reaction of ethyldiazoacetate (EDA) 1 in toluene solvent (0.15M), at 60 °C for 24 h. Error bars account for a 5% uncertainty.

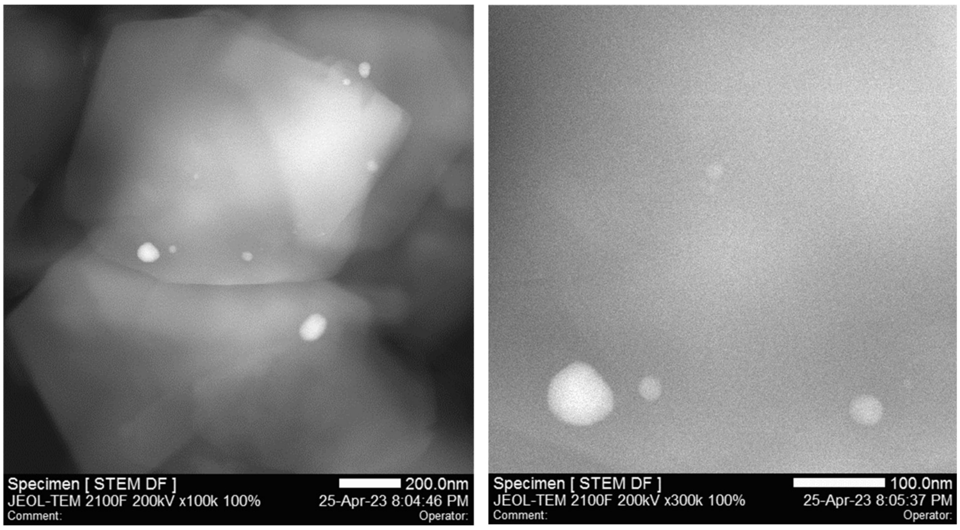

Figure S26. High resolution transmission electron microscopy (HR–TEM) images of Ag–LiNaY after reaction in Figure S21.
